# Pyrene‐Conjugated, 2‐Pyridinecarboxaldehyde Derivatives as N‐Terminus‐Specific Tags for MALDI‐ and LALDI‐MS

**DOI:** 10.1002/rcm.70034

**Published:** 2026-01-20

**Authors:** Mujia Jenny Li, Murat Kucukdisli, Florian Braun, David W. Will, Nadine Meier, Bettina Wehrle, Larissa Chiara Meyer, Melanie Christine Föll, Oliver Schilling

**Affiliations:** ^1^ Institute for Surgical Pathology, Faculty of Medicine University Medical Centre Freiburg, University of Freiburg Freiburg Germany; ^2^ Institute for Pharmaceutical Sciences University of Freiburg Freiburg Germany; ^3^ Chemical Synthesis Core Facility, European Molecular Biology Laboratory Heidelberg Germany; ^4^ Faculty of Biology University of Freiburg Freiburg Germany; ^5^ German Cancer Consortium (DKTK), German Cancer Research Center (DKFZ) Heidelberg Germany

## Abstract

**Background:**

Proteolytic processing is a fundamental post‐translational modification. Liquid chromatography–tandem mass spectrometry (LC–MS/MS) workflows are powerful for degradomic analyses but inherently sacrifice spatial information, a critical aspect for investigating biological systems such as aberrant extracellular matrix remodeling and alterations of the tumor microenvironment. Matrix‐assisted laser desorption/ionization (MALDI) offers potential for fast spatial profiling, but MALDI imaging of tryptic peptides is still challenged by spectral crowding and restricted abilities for MALDI MS/MS identification.

**Methods:**

To address these limitations, we developed pyrene‐conjugated 2‐pyridinecarboxaldehyde (pyr‐2PCA) tags for selective N‐terminal labeling and enhanced detection sensitivity. The 2PCA reagent exclusively modifies N‐terminal α‐amines, not lysine ε‐amines, as could be confirmed in MALDI‐MS, with concentration‐dependent side reactions minimized by dilution. A distinct reporter ion produced by 2PCA‐labeled peptides in prm‐PASEF (MALDI MS/MS) serves as a unique marker for successful labeling.

**Results:**

The covalent conjugation of 2PCA with a pyrene structure results in the pyr‐2PCA tag that enables matrix‐free, label‐assisted laser desorption ionization mass spectrometry (LALDI‐MS) measurements of peptides. We demonstrate that labeling with a pyrene‐coupled 2PCA tag (pyr‐2PCA) prior to tryptic digestion results in the selective detection of N‐terminal peptides in LALDI, with no significant off‐target labeling.

**Conclusions:**

This study presents the first presentation and characterization of this novel pyr‐2PCA tag, thereby laying the groundwork and demonstrating its future potential for MALDI/LALDI‐based in situ spatial N‐terminomics to study proteolytic processes.

**Abbreviations**
2PCA: 2‐pyridinecarboxaldehyde
Ac‐: acetylated N‐terminus
ACN: acetonitrile
CHCA: α‐cyano‐4‐hydroxycinnamic acid
DMSO: dimethyl sulfoxide
FA: formic acid
FFPE: formalin‐fixed paraffin‐embedded
iprm‐PASEF: imaging parallel reaction monitoring‐parallel accumulation serial fragmentation
ITO: indium tin oxide
LALDI: label‐assisted laser desorption/ionization
LC–MS/MS: liquid chromatography–tandem mass spectrometry
*m*/*z*
: mass‐to‐charge ratio
MALDI: matrix‐assisted laser desorption/ionization
MS: mass spectrometry
MSI: mass spectrometry imaging
NHS: *N*‐hydroxysuccinimide
pip2PCA: 6‐(piperazin‐1‐ylmethyl)picolinaldehyde hydrochloride
prm: parallel reaction monitoring
pyr‐2PCA: 6‐((4‐(4‐(pyren‐1‐yl)butanoyl)piperazin‐1‐yl)methyl)picolinaldehyde
SPE: solid‐phase extraction
SIMSEF: spatial ion mobility‐scheduled exhaustive fragmentation
PASEF: parallel accumulation serial fragmentation
RMS: root mean square
TFA: trifluoroacetic acid
TIMS: trapped ion mobility spectrometry
TMPP: tris (trimethoxyphenyl)phosphonium
1/*K*_0_
: inverse reduced ion mobility

## 1 Introduction

Protein N‐termini hold valuable information about the functional status of proteins. Proteolytic processing is a quasi‐irreversible post‐translational modification that influences a protein's localization, stability, activity, and folding. It yields truncated proteins as cleavage products that possess neo‐N‐termini. Dysregulated proteolytic cleavage can contribute to pathological conditions and plays an important role in disease‐related processes including aberrant extracellular matrix remodeling and alterations of the tumor microenvironment [1, 2, 3, 4, 5, 6]. The clinical success of protease inhibitors, particularly in antiviral therapy, underscores the therapeutic potential of targeting proteolytic pathways and the importance of understanding proteolytic processes in biological systems [7, 8, 9, 10]. Neo‐epitope antibodies are entering diagnostics to monitor proteolytic cleavage products, for example, in fibrotic remodeling [11].

The use of liquid chromatography–tandem mass spectrometry (LC–MS/MS)‐based workflows enables deep characterization of proteolytic cleavage events. Most N‐terminomic approaches employ N‐terminal labeling and enrichment steps, thereby distinguishing between native or neo‐ and tryptic N‐termini [12, 13, 14, 15, 16]. However, most labeling reagents are based on N‐hydroxysuccinimide (NHS) ester modifications that predominantly are unable to modify only the α‐amine while leaving the ε‐amines on lysine side chains unaltered. An exception is tris (trimethoxyphenyl)phosphonium (TMPP) introduced via an NHS group (TMPP‐Ac‐OSu), which can enable selective α‐amine tagging for LC–MS/MS N‐terminomics but requires strict pH control to 8.2 for selectivity [17, 18]. TMPP‐Ac‐OSu was also used to label peptides for MALDI MS/MS analysis, demonstrating enhanced sequence‐informative fragmentation by the fixed‐charge tag [19]. MacDonald et al. introduced the reagent 2‐pyridinecarboxaldehyde (2PCA), which reacts exclusively with the N‐terminal α‐amine through an intramolecular cyclization, forming a stable imidazolidinone ring that cannot emerge with lysine side‐chain amines [20]. Hence, the 2PCA reaction can exclusively modify peptides that are native or neo‐N‐terminal if labeling is performed prior to tryptic digestion.

Because all of the protein‐level labeling methodologies rely on bulk protein extraction and LC–MS/MS measurements, most spatial information and tissue context are lost during sample preparation. Matrix‐assisted laser desorption/ionization (MALDI)‐based approaches enable rapid in situ probing of peptides and proteins by MS/MS, hence in an antibody‐independent manner. For spatial proteomics, typical MALDI imaging workflows employ trypsinization. However, MALDI imaging of tryptic peptides is still limited by two major issues: first, the lack of automatic MS/MS‐based identification of every detected signal and second, the missing chromatographic separation and therefore high complexity of the sample at each laser shot. Recently, several trapped ion mobility exploiting tools such as iprm‐PASEF or SIMSEF paved the way for integrating simple, automated, and standardized MS/MS analyte verification into MALDI imaging [21, 22, 23, 24, 25]. Here, trapped ion mobility spectrometry (TIMS) is utilized as an additional dimension to accumulate and separate ions, thereby enabling a multiplexed fragmentation and more efficient ion usage. However, the missing chromatographic separation and the inability to specifically target low abundant signals in MALDI still remain as hurdles to overcome. This limitation is particularly relevant for MALDI imaging‐based N‐terminomics, because N‐terminal peptides represent only one of often numerous tryptic peptides per protein, hence being severely underrepresented.

Label‐assisted laser desorption/ionization mass spectrometry (LA‐LDI‐ or LALDI‐MS) is a matrix‐free technique in which the analyte is coupled to a polyaromatic tag by a covalent bond or chelation. The label attachment leads to analyte desorption and ionization without the need for co‐crystallization with an external matrix. It is proposed that laser energy is absorbed via its intrinsic π‐electron system and ionization occurs via an electron transfer process [26, 27]. Pyrene‐derived structures have proven particularly effective in LALDI‐MS, enabling efficient ionization and selective detection of labeled molecules in a complex mixture [28, 29]. To date, LALDI‐MS has been used for evaluating chemical reaction mechanisms and detection assays for metal ions, 1,2‐diols, and primary amines [28, 30, 31]. Importantly, Yoneda et al. used amidopyrene NHS ester derivatives to tag proteins at their lysine side chain ε‐amines before tryptic digestion and demonstrated increased detectability of tagged peptides in LALDI‐MS, showcasing the potential of covalently tagged pyrene structures to boost peptide signal intensity [32].

Here, we present pyrene‐coupled 2PCA tags designed to selectively label protein N‐termini and neo‐N‐termini via a cyclic reaction with α‐amines. By integrating a pyrene structure into the 2PCA tag, we open the perspective to specifically tag and detect N‐terminal peptides in a LALDI‐based manner. We provide a first characterization of the pyrene‐incorporating 2PCA tags and their application for N‐terminal peptide labeling in LALDI/MALDI‐MS, thereby laying the groundwork and demonstrating future potential for LALDI/MALDI‐based in situ spatial N‐terminomics to study proteolytic processes.

## 2 Materials and Methods

### 2.1 Compound Synthesis

Reagents were purchased from Sigma‐Aldrich, BLD pharm, and TCI Chemicals and used without further purification. All solvents, including anhydrous solvents, were used as obtained from the commercial sources. Air‐ and water‐sensitive reagents and reactions were generally handled under argon atmosphere. The reaction progress was monitored by TLC on Merck silica gel plates 60 F254. Detection was executed with a UV cabinet HP‐UVIS (biostep) at 254 nm or with potassium permanganate staining. Flash chromatographic purification was performed on a Biotage Isolera One purification system using Biotage Sfär Silica D and Biotage SFär C18 D flash cartridges, respectively. Nuclear magnetic resonance spectra were recorded on a Bruker Avance (400 MHz) NMR spectrometer (Bruker Biospin, Ettlingen, Germany) at 298 K. Chemical shifts (δ) are given in parts per million (ppm), coupling constants (J) given in Hertz (Hz), and multiplicity is reported using standard abbreviations. The following chemical shifts were detected in the ^1^H NMR (400 MHz, DMSO‐*d*_6_) spectrum of pyr‐2PCA: δ 9.97 (d, J = 0.8 Hz, 1H), 8.44 (d, J = 9.3 Hz, 1H), 8.29–8.24 (m, 2H), 8.23 (d, J = 1.7 Hz, 1H), 8.21 (d, J = 3.3 Hz, 1H), 8.16–8.09 (m, 2H), 8.08–8.00 (m, 2H), 7.94 (d, J = 7.8 Hz, 1H), 7.83 (dd, J = 7.7 Hz, 1.1 Hz, 1H), 7.75 (dd, J = 7.8 Hz, 1.2 Hz, 1H), 3.72 (s, 2H), 3.54–3.48 (m, 1H), 3.46–3.40 (m, 1H), 3.37–3.29 (m, 2H), 2.47 (t, J = 7.2 Hz, 2H), 2.43–2.39 (m, 4H), 2.05–1.95 (m, 2H) ppm. For dma‐pyr‐GG, the ^1^H NMR (400 MHz, CD_3_OD) spectrum showed the following chemical shifts: δ 8.50 (d, J = 9.3 Hz, 1H), 8.40–8.37 (m, 4H), 8.28 (d, J = 7.9 Hz, 1H), 8.18 (d, J = 9.3 Hz, 1H), 8.04 (d, J = 7.9 Hz, 1H), 3.96–3.93 (m, 4H), 3.64 (s, 6H), 3.46–3.38 (m, 2H), 2.48 (t, J = 7.2 Hz, 2H), 2.24–2.08 (m, 2H). UHPLC: t_*R*_ = 1.83 min, MS (ESI): 476 (M + H)^+^. UHPLC/MS analyses were performed on Agilent 1290 series equipment consisting of an Agilent 1290 quaternary pump, a 1290 sampler, a 1290 thermostated column compartment, and a 1290 Diode array detector VL+ equipped with a quadrupole LC/MS 6120 and an Infinity 1260 ELSD. The analytical column used was a Titan C18 UHPLC Column (2.1 × 30 mm, 1.9 μm) operated at 40°C and 1.5 mL/min flow rate with a gradient (10%–15% B in 0.4 min, 15%–100% B in 1.6 min, and 100% B for 0.5 min) using water (A) and acetonitrile (ACN) (B), both containing 0.1% trifluoroacetic acid (TFA), as solvents. Compound purity was determined by ELSD monitoring. 6‐(Piperazin‐1‐ylmethyl)picolinaldehyde hydrochloride (pip2PCA) was synthesized following the literature procedure [20]. A mixture of commercial available 1‐pyrenebutyric acid (58 mg, 0.20 mmol), 6‐(piperazin‐1‐ylmethyl)picolinaldehyde hydrochloride (pip2PCA) (48 mg, 0.20 mmol), 1‐[bis (dimethylamin)methylen‐1H‐1,2,3‐triazol[4,5‐b] pyridinium‐3‐oxid‐hexafluorophosphate (HATU, 91 mg, 0.24 mmol), and triethyl amine (0.14 mL, 0.60 mmol) in anhydrous DMF (1.0 mL) was reacted at room temperature overnight. After completion of the reaction, the solvent was reduced, and the crude material was purified on a Biotage SFär C18 D‐12 g column using a water/methanol gradient to obtain 49 mg (0.10 mmol, 52%) of the analytically pure compound 6‐((4‐(4‐(pyren‐1‐yl)butanoyl)piperazin‐1‐yl)methyl)picolinaldehyde (pyr‐2PCA). Dma‐pyr‐GG was synthesized from commercially available 4‐(pyren‐1‐yl)butanoic acid in several synthesis steps as shown in Figure S3. A spontaneous oxidation of the di‐amino group, leading to a mass addition of +16 Da, was observed upon shipping and short‐term storage. The species +16 Da remained stable and showed no further reactions or degradation. All used compounds are summarized with all used compounds in the Supporting Information (Table S1).

### 2.2 Tissue and Peptide Samples

Mouse kidney tissues were obtained as surplus tissue from animals that had been sacrificed for unrelated experimental purposes in accordance with the approved animal experimentation protocols. The mouse kidney samples represent leftover material that would have been discarded otherwise. Mouse kidney samples were formalin‐fixed and paraffin‐embedded (FFPE) according to standard protocols [33]. FFPE blocks were sliced into 2‐μm‐thick sections and mounted onto IntelliSlides (Bruker Daltonics, Bremen, Germany). The synthetic peptides were obtained from different sources, and a summary can be found in the Supporting Information (Table S1).

### 2.3 In‐Solution Pip2PCA Labeling

In‐solution labeling was carried out in a reaction volume of 15 μL containing 300 μM peptide, 15 mM pip2PCA (stock in DMSO) yielding a molar ratio pip2PCA reagent to peptide of 50:1, 28.5% DMSO, and pH of 8 by adding 25 mM phosphate buffer. Labeling was carried out for 24 h at 37°C. After the labeling reaction, the reaction mixture was diluted 1:100 (V/V) with 1% formic acid (FA) in H_2_O and incubated at 37°C for 5 min. Then, 1 μL of sample was spotted directly on indium tin oxide (ITO) slides (Bruker Daltonics, Bremen, Germany), mixed 1:1 (V/V) with 10 mg/mL CHCA matrix (50% ACN, 1% TFA), and dried in an oven at 50°C for further mass spectrometry analysis.

### 2.4 In‐Solution Pyr‐2PCA Labeling

For pyr‐2PCA, 300 μM peptide was labeled with 3 mM pyr‐2PCA (10:1 M ratio) in a reaction volume of 30 μL. The DMSO amount was 7.1%, and 50% ACN was added for solubility. The pH was adjusted to 8 using phosphate buffer, yielding a final concentration of 6.7 mM. The labeling reaction was carried out for 24 h at 37°C. Afterwards, samples were filled up to 200 μL with 1% TFA in H_2_O, incubated at 37°C for 5 min, and purified using HyperSep SpinTip C‐18 Columns for Solid‐Phase Extraction (SPE) (Thermo Fisher Scientific, Waltham, USA). In case of peptide spike‐ins, synthetic peptides were added directly before Hypersep cleanup (4.5 nmol each).

### 2.5 In‐Solution Tryptic Digestion

For tryptic digestion, a reaction mixture of 30 μL was prepared as in 2.4 (or 15 μL as in 2.3) and diluted with 1% TFA to a final volume of 200 μL and loaded on Hypersep SpinTip C‐18 columns (Thermo Fisher Scientific, Waltham, USA). After three washes with 8% ACN and 0.05% TFA, peptides were eluted using 68% ACN and 0.05% TFA. Samples were vacuum‐dried and resuspended in 50 μL of 100 mM HEPES. Afterwards, 0.5 μg of Trypsin Gold (Promega, Madison, USA) was added and incubated for 2 h at 50°C while shaking. Afterwards, samples were cleaned up again using Hypersep for Solid‐Phase Extraction (SPE) as described above. The elution mixture (50 μL) was spotted directly on ITO slides, mixed 1:1 (V/V) with CHCA matrix (Sigma‐Aldrich, Munich, Germany) (10 mg/mL, 50% ACN, 1% TFA), and dried in an oven at 50°C for 5 min for further mass spectrometry analysis.

### 2.6 Dma‐Pyr‐GG Titration

Titration was carried out with a consistent amount of unlabeled synthetic peptides MRFA (SP3), Ac‐AVRPGYP‐Lys (Ac)‐OH, H‐AVRPGYP‐Lys (Ac)‐OH, Ac‐AVAPGYPA‐OH, and Ac‐AVRPGAPA‐OH of 1.123 pmol (aqueous stock solutions) of each per spot. Dma‐pyr‐GG was mixed to it in five different amounts: 2.25 nmol (pure), 22.4 pmol (dil1), 11.23 pmol (dil2), 7.49 pmol (dil3), 5.62 pmol (dil4), and 4.49 pmol (dil5). One spot was prepared for each condition on the same ITO slide and imaged in one single imaging run. After LALDI measurement, MALDI measurements were performed as described in Section 2.8.

### 2.7 Pip2PCA Labeling Analysis With MALDI‐MS, MALDI‐MS/MS, and MALDI prm‐PASEF

All measurements were carried out on a TimsTOF FleX (Bruker Daltonics, Bremen, Germany) in positive reflector mode. The instrument is equipped with a Nd:YAG laser emitting at 355 nm. MALDI MS1 spot measurements were performed with TIMS turned off, MALDI Plate Offset 80 V, Funnel 1 RF 250 Vpp, Funnel 2 RF 500 Vpp, Deflection 1 Delta 80 V, Multiple RF 500 Vpp, Collision RF 2500 Vpp, Transfer Time 180 μs, and Pre Pulse Storage 20 μs. The mass range was set between *m*/*z* 500 and 3500 and partially restricted individually depending on the analyte mass. The laser was set to 600 shots with 10 000 Hz, Imaging 50 μm as laser setup, 5 random moves per grid, and 200 shots at raster position. All MALDI MS/MS measurements were performed in prm‐PASEF mode, with a mass range of *m*/*z* 50–1700 and a 1/*K*_0_ range of 1.2–2.23 V*s/cm^2^, utilizing a ramp time of 230 ms. Tuning settings were set as in MALDI‐MS1 except Funnel 1RF 500 Vpp, Collision RF 1000 Vpp, Transfer Time 55 μs, Pre Pulse Storage 5 μs, Collision Energies from *m*/*z* 500–1500 to 55–105 eV, and *m*/*z* width 1. The laser was set to 2000 shots with 10 000 Hz with 5 bursts, Imaging 50 μm as laser setup, 5 random moves per grid, and 200 shots at raster position. The prm‐PASEF measurements were analyzed and plotted in DataAnalysis (Bruker Daltonics, Bremen, Germany). MS2 spectra were exported as .mgf files, and assigned theoretical b‐ and y‐ions (tolerance +/− 0.3 Da) were visualized using the pyteomics package in Python [34]. For all experiments, five replicate spectra were acquired for every single condition, and one representative was chosen for visualization. The iprm‐PASEF measurements of the reporter ion were carried out with 2000 number of laser shots with 5000 Hz, high sensitivity mode turned on, Imaging 50 μm as laser setup, and two isolation windows of 1.4–1.72 V*s/cm^2^ with *m*/*z* 1300 and 1.76–1.9 V*s/cm^2^ with *m*/*z* 1900. Mass width was set to *m*/*z* 80 and collision energies from *m*/*z* 1000–2000 to 100–180 eV. Collision RF was set to 1200 Vpp, Transfer Time 80 μs, and Pre Pulse Storage 10 μs.

### 2.8 Pyr‐2PCA Analysis in MALDI/LALDI Imaging Mode

All measurements of pyr‐2PCA were carried out in imaging mode to account for laser shot and intra‐spot variability. Additionally, LALDI and MALDI measurements were performed on the same spots, meaning that MALDI measurements were carried out on those spots that were measured in LALDI and were overspotted with 1 μL of CHCA matrix (10 mg/mL, 50% ACN, and 1% TFA) after measurement. Identical measurement regions were selected in FlexImaging. MALDI and LALDI MS1 measurements for SP3 and dma‐pyr‐GG were carried out with the following settings: MALDI Plate Offset 50 V, Funnel 1 RF 250 Vpp, Funnel 2 RF 500 Vpp, Deflection 1 Delta 70 V, Multiple RF 500 Vpp, Collision RF 2000 Vpp, Transfer Time 150 μs, and Pre Pulse Storage 15 μs. For dma‐pyr‐GG, the mass range was set from *m*/*z* 250 to 1500, and for SP3, it was set from *m*/*z* 250 to 1700. The laser was set to 200 shots with 10 000 Hz and Imaging 50 μm as laser setup. MALDI MS1 measurements of β‐amyloid were adjusted for a higher *m*/*z* range, resulting in the following measurement parameters: *m*/*z* 300–2500, MALDI Plate Offset 50 V, Funnel 1 RF 250 Vpp, Funnel 2 RF 500 Vpp, Deflection 1 Delta 70 V, Multiple RF 500 Vpp, Collision RF 2000 Vpp, Transfer Time 180 μs, and Pre Pulse Storage 20 μs. The laser was set to 500 shots with 10 000 Hz and Imaging 50 μm as laser setup. For LALDI measurements, the exact same measurement settings were used, except that “High Sensitivity Detection ‐ Low Sample Amount” and “Focus Mode” were turned on. The iprm‐PASEF measurements were carried out with the following parameters: *m*/*z* 300–2500, 1/*K*_0_ range 1.00–2.00 V*s/cm^2^, ramp time 230 ms, MALDI Plate Offset 80 V, Funnel 1 RF 500 Vpp, Funnel 2 RF 500 Vpp, Deflection 1 Delta 80 V, Multiple RF 500 Vpp, Collision RF 750 Vpp, Transfer Time 55 μs, Pre Pulse Storage 8 μs, “High Sensitivity Detection ‐ Low Sample Amount” on, collision energies from *m*/*z* 500–1500 to 55–105 eV, and m/z width 1. The laser was set to 1000 shots with 10 000 Hz and Imaging 50 μm as laser setup. All imaging datasets were analyzed and visualized in SCiLS Lab (Bruker Daltonics, Bremen, Germany). Root mean square (RMS) normalization and an *m/z* interval width of 10 ppm were selected for all analyses.

### 2.9 On Slide Pip2PCA Tissue Labeling

For MALDI imaging of the reporter ion in iprm‐PASEF mode, one FFPE slide of mouse kidney was first deparaffinized in a series of xylol and ethanol/water dilutions as described previously and then washed with 10 mM HEPES for 5 min. Antigen retrieval was performed in a citric acid monohydrate buffer with pH 6.0 in a steamer for 1 h at approximately 100°C and washed twice with 10 mM HEPES for 5 min. Then, labeling reagent was applied with 10 mM pip2PCA and 165 mM phosphate buffer pH 8 and incubated in a humidified chamber at 37°C for 24 h. Afterwards, the slide was washed with 2% FA four times for 10 min and 70% ACN three times for 1 min and incubated in 10 mM ammonium bicarbonate (AmBC) solution for 30 min. The slide was air‐dried, and freshly prepared N‐tosyl‐L‐phenylalanine chloromethyl ketone–treated trypsin (Worthington, Lakewood, NJ, USA) (0.1 mg/mL in 10% ACN, 40 mM AmBC) was sprayed using the M3+ sprayer (HTX Technologies, Chapel Hill, NC, USA) with the following parameters: temperature 30°C, nozzle velocity 750 mm/min, flow rate 30 μL/min, number of passes 8, track spacing 2 mm, pattern CC, drying time 0 s, and nitrogen gas pressure set to 10 psi. The slide was then incubated in a humidified chamber for 2 h at 50°C. CHCA matrix (Sigma‐Aldrich, Munich, Germany) (10 mg/mL, 70% ACN, 0.1% TFA, 7 mM ammonium phosphate) was applied using the M3+ sprayer with the following parameters: temperature 60°C, nozzle velocity 1350 mm/min, flow rate 100 μL/min, number of passes 8, track spacing 3 mm, pattern CC, drying time 10 s, and nitrogen gas pressure set to 10 psi.

### 2.10 Light Microscopy

Light microscopy images were acquired using a LEICA EC3 microscope and a 4×/0.10 objective (Leica Microsystems, Wetzlar, Germany). Images were saved using the LAS EZ 3.4 software (Leica Microsystems, Wetzlar, Germany).

### 2.11 Use of Large Language Models, AI, and Machine Learning Tools

“OpenAI” and “DeepL Write” were used to improve language, supporting literature search and writing style.

## 3 Results

### 3.1 Detectability of Pip2PCA‐Labeled Peptides by MALDI‐MS

**FIGURE 1:**
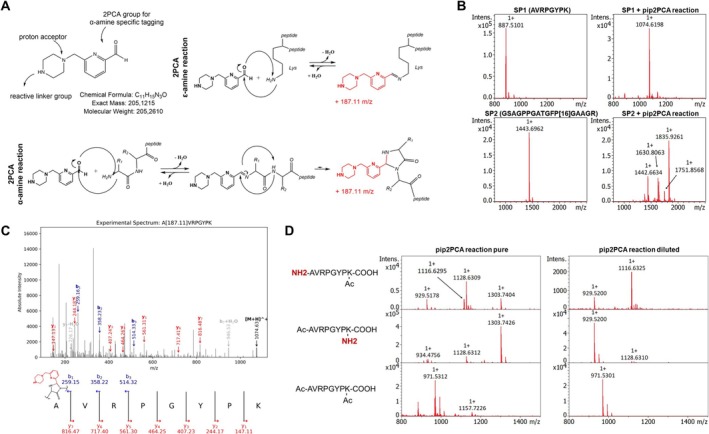
Pip2PCA‐labeling detected in MALDI‐MS. (A) Pip2PCA compound structure and reaction mechanisms for α‐ and ε‐amine reactions. (B) Representative MALDI MS1 spectra of unlabeled and labeled synthetic peptides SP1 and SP2, showing successful labeling by a mass shift of *m*/*z* 187.11. Figure S1C includes a zoomed spectrum of SP1 + pip2PCA reaction. The reaction mixture was diluted 1:100 with 1% FA in H_2_O before spotting and MALDI‐MS measurement. (C) MS2 spectrum of SP1 acquired with MALDI MS/MS, highlighting detected b‐ and y‐ions. The sequence at the bottom annotates the b‐ and y‐ions with their theoretical *m*/*z*. (D) MALDI MS1 spectra of three differently acetylated SP1‐based species, showing the measurement of the pure reaction mixture on the left side and the 1:100 dilution with 1% FA in water on the right side.

The chemical reaction of 2PCA with primary α‐amines has been extensively characterized in previous studies using LC‐ESI‐MS/MS and NMR spectroscopy, demonstrating both its efficiency and selectivity over lysine ε‐amino side chains (Figure 1A) [20, 35, 36, 37, 38]. In contrast, MALDI‐MS typically produces singly charged ions and is prone to cluster formation [39, 40, 41, 42, 43]. Therefore, the initial objective of this study was to evaluate the feasibility, detectability, and transferability of 2PCA‐based N‐terminal labeling for MALDI‐MS analysis.

To clearly evaluate 2PCA labeling in MALDI‐MS, we chose synthetic peptides as an experimental setup with low complexity. To allow subsequent coupling with functional groups such as a pyrene structure, a piperazine‐coupled 2PCA derivative (pip2PCA) was used for all experiments. Following established protocols, the reaction was carried out at 37°C and pH 8 with a 1:50 M ratio of peptide to pip2PCA for 24 h [20, 35, 36, 37, 38]. Under these conditions, labeling efficiencies exceeded 80% for the synthetic peptide AVRPGYPK (SP1), as determined by MALDI‐MS (Figure 1B). Correct labeling at the N‐terminus was confirmed by MALDI MS/MS analysis of two synthetic peptides (Figure 1C (SP1) and Figure S1 (DNIQGITKPAIR)). The MS/MS spectrum for SP1 displayed an unshifted y‐ion series, while, for example, the b1‐ to b3‐ion series exhibited a mass shift of *m*/*z* 187.11, consistent with pip2PCA modification. Notably, we observed that pip2PCA labeling influenced the fragmentation pattern compared to the unlabeled peptide (Figure S1). Additionally, we observed a double‐labeling side reaction that is labile under aqueous conditions, as further elaborated below. However, peaks corresponding to side products arising from pip2PCA double labeling (e.g., *m*/*z* 1261.73 and *m*/*z* 1279.74 for SP1) remained below 3% relative intensity (Figure S1) upon aqueous dilution.

For the synthetic peptide GSAGPPGATGFPGAAGR (SP2) comprising a glycine at the N‐terminal position, several unexpected signals and a lower labeling efficacy were observed consistent with previous reports (Figure 1B) [20]. Two specific peaks at *m*/*z* 1630.8063 and 1835.9261 suggest a two‐step reaction mechanism consisting of an initial condensation followed by a secondary pip2PCA addition without water loss. Although the exact structure remains unresolved, we highlight that a similar, aldehyde‐involving double addition has been observed with formaldehyde cross‐linking by Tayri‐Wilk et al. [44]. We propose that both observations share an analogous reaction mechanism.

Detection of pip2PCA‐labeled peptides by LC–MS/MS includes, by default, a chromatographic separation step that is typically based on reversed‐phase chromatography in acidic conditions. In potential MALDI applications, this step is missing. Hence, we directly (without clean‐up by chromatography or solid‐phase extraction (SPE)) spotted the labeling reaction mixture on ITO slides with subsequent application of the MALDI matrix and MALDI‐MS measurement. For both tested peptides SP1 and SP2, this approach yielded several unexpected *m*/*z* values, indicating multiple pip2PCA‐labeled products rather than a single N‐terminally labeled molecular specimen.

To better understand the reactivity of pip2PCA toward ɑ‐ and ε‐amines in this context, the synthetic peptide SP1 containing a C‐terminal lysine was synthesized with (i) N‐terminal acetylation only, (ii) lysine side chain acetylation only, or (iii) acetylation of both primary amines. These peptide species were subjected to the “direct spotting” approach described above. For the peptide species with free α‐ or ε‐amines, we observed unexpected mass shifts at *m*/*z* 199.11 (+12.00 Da relative to the expected pip2PCA condensation product) and *m*/*z* 374.22 (consistent with a double pip2PCA condensation) (Figure 1D). Following a 1:100 dilution with 1% FA in water, only the peptide with free α‐amines exhibited a mass shift, this being the anticipated shift of *m*/*z* 187.11. This result confirms the selective and correct labeling of the N‐terminus by pip2PCA and its detectability by MALDI‐MS. It also highlights the necessity of an acidic‐aqueous incubation step for the hydrolysis of intermediate adducts, a step that is not necessarily included in MALDI‐MS (as opposed to LC‐ESI‐MS/MS).

Therefore, we conclude that the observed non‐canonical mass shifts result from reactions involving a single primary amine, with double labeling occurring on the same amine moiety rather than multiple, distinct sites. We hypothesize that elevated concentrations of pip2PCA promote side reactions, potentially involving the pip2PCA‐coupled piperazine linker as a reactive site. The structural identity of the *m*/*z* 199.11 mass shift remains elusive. However, these side reactions appear to be concentration dependent and reversible and can be minimized under dilute, aqueous conditions.

### 3.2 Protein‐Level Labeling by Pip2PCA in MALDI‐MS

#### 3.2.1 In‐Solution Labeling for MALDI‐MS

Our ultimate objective was to selectively target neo‐N‐termini for MALDI‐MS while avoiding labeling (and possible detection) of internal tryptic peptides that are generated post‐labeling upon trypsinization. Therefore, efficient removal of the unreacted, excess pip2PCA compound is crucial after the labeling reaction. Pip2PCA labeling of proteins was demonstrated for LC‐ESI‐MS/MS by MacDonald et al. [20]. However, the previously presented data on acid‐labile side‐products detected by MALDI‐MS motivated us to evaluate the workflow using MALDI‐MS detection of peptides.

**FIGURE 2:**
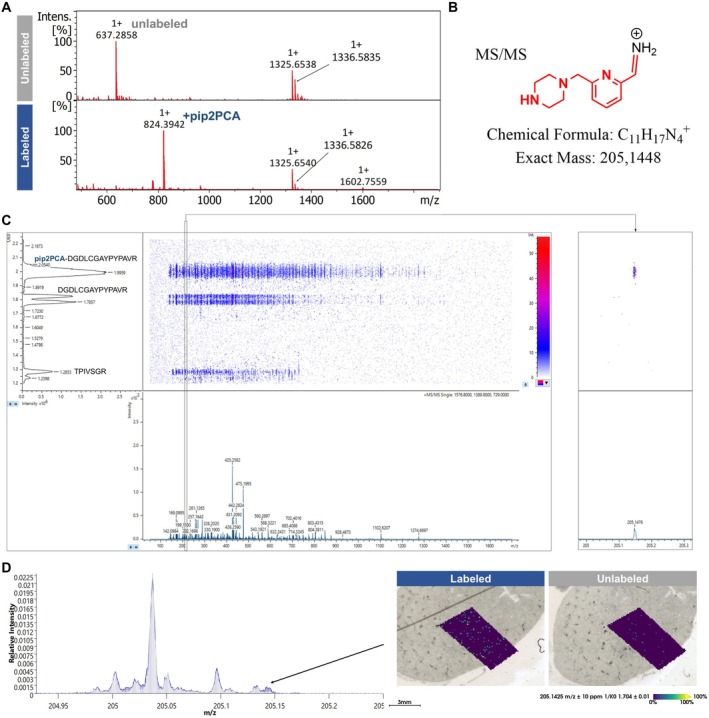
Protein‐level labeling by pip2PCA. (A) Representative MS1 spectra of tryptic digested β‐amyloid (DAEF**R**HDSGYEVHHQ**K**LVFFAEDVGSN**K**GAIIGLMVGGVVIA), labeled and unlabeled with pip2PCA. Marked inside the mass spectra are the respective *m*/*z* values of either the unlabeled N‐terminal peptide or the respective pip2PCA‐labeled species. (B) Proposed structure of detected reporter ion after successful N‐terminal pip2PCA labeling, resulting in the mass of M*+*H^+^ of *m*/*z* 205.14. (C) Single prm‐PASEF measurement of a spot of labeled and tryptic digested peptide DGDLCGAPYPAVRAAGPKTPIVSGR. DGDLCGAPYPAVR exhibits a mass shift of + *m*/*z* 56.08 because of a tert‐butyl protection group. Three isolation windows were chosen, targeting pip2PCA‐DGDLCGAPYPAVR+tert‐butyl (*m*/*z* 1576.7944), DGDLCGAPYPAVR+tert‐butyl (*m*/*z* 1389.6838), and TPIVSGR (*m*/*z* 729.4256). Only the trace of the labeled N‐terminal peptide *m*/*z* 1576.7944 exhibited a signal at *m*/*z* 205.1476 that is named reporter ion in the course of this study. (D) iprm‐PASEF imaging run of a FFPE mouse kidney tissue that was labeled with pip2PCA on slide before tryptic digestion. The diagram shows a zoomed overlay of the RMS‐normalized mean spectra of the labeled tissue specimen (blue) and unlabeled control (gray), including the respective ion images of *m*/*z* 205.1425.

We used the β‐amyloid polypeptide DAEF**R**HDSGYEVHHQ**K**LVFFAEDVGSN**K**GAIIGLMVGGVVIA with three tryptic cleavage sites for evaluation. MALDI‐MS of the trypsinized, unmodified polypeptide showed strong signals for the sequences DAEFR (*m*/*z* 637.2940; N‐terminus), HDSGYEVHHQK (*m*/*z* 1336.6029), and LVFFAEDVGSNK (*m*/*z* 1325.6735) (Figure 2A). The C‐terminal peptide GAIIGLMVGGVVIA (*m*/*z* 1269.7598) remained undetected, possibly because of the lack of a lysine or arginine residue. After the pip2PCA labeling, the reaction mixture was diluted and cleaned up using C18 SPE, including washes with 0.05% TFA and 8% ACN. Afterwards, the pip2PCA‐labeled polypeptide was trypsinized for 2 h at 50°C and analyzed using MALDI‐MS. The signal of the two internal tryptic peptides HDSGYEVHHQK (*m*/*z* 1336.6029) and LVFFAEDVGSNK (*m*/*z* 1325.6735) remained unaffected. The signal of the native N‐terminal peptide DAEFR (*m*/*z* 637.2940) disappeared, and a new signal for pip2PCA‐DAEFR *(m*/*z* 824.4049) could be detected. Hence, we successfully reproduced pip2PCA labeling of polypeptide N‐termini with MALDI‐MS detection.

#### 3.2.2 Reporter Ion Detection in prm‐PASEF (MALDI MS/MS) Mode

Recently, prm‐PASEF has been introduced as a novel multiplexed MS/MS acquisition mode for the TimsTOF Flex. Here, a dual trapped ion mobility mass spectrometry (TIMS) cell setup before the quadrupole allows the accumulation and separation of several precursors by their inverse reduced ion mobility (1/*K*_0_). Thereby, ion usage is optimized, leading to improved tandem mass spectrometry data generation in MALDI imaging and facilitating in situ identification of peptides. For pip2PCA‐labeled peptides, MALDI MS2 using prm‐PASEF highlighted a reporter ion of *m*/*z* 205.1476 in their MS/MS fragmentation trace (Figure 2B,C). The detected fragment at *m*/*z* 205.1476 fits the expected *m*/*z* of a fragment ion derived from the imidazolidinone ring structure, particularly an iminium ion as shown in Figure 2B, at *m*/*z* 205.1448 with a mass offset of approximately 13.6 ppm. Consistent with this assignment, a corresponding fragment at *m*/*z* 475.25 was observed in iprm‐PASEF mode using the modified pip2PCA label described below (Section 3.3, Figure 4B).

When analyzing a mixture of pip2PCA‐labeled and unlabeled peptides, MS2 reporter ion detection was sufficient to determine the pip2PCA labeling status, regardless of any assumed MS1 mass shift (Figure 2C). We propose that the presence of this reporter ion in prm‐PASEF or MS/MS measurements is of value as a marker for pip2PCA labeling. We successfully demonstrated in situ detection of the described reporter ion in a MALDI imaging measurement of a pip2PCA‐labeled tissue. Here, an FFPE mouse kidney tissue was prepared as described in Section 2.9. For iprm‐PASEF acquisition, two broad, exploratory isolation windows were employed, and signal resolution was focused on the lower *m*/*z* range. The use of these wide windows enabled fragmentation of a heterogeneous population of peptides, providing evidence for partially successful pip2PCA labeling within the selected *m*/*z* range. Here, we report the detection of a signal at *m*/*z* 205.1424, matching the expected reporter ion mass with an error of 11.7 ppm (Figure 2D). This signal was detected at low intensity for the pip2PCA‐labeled sample, whereas it was only found at the noise level in the control. We hypothesize that this signal originates from the pip2PCA tag, although its exact identity has yet to be fully validated. Proteome‐wide studies with regard to a putative sequence specificity of the proposed reporter ion, as well as its detectability in LC‐ESI‐MS/MS, remain beyond the scope of the present proof‐of‐concept study.

### 3.3 2PCA Labeling in LALDI by Integration of Pyrene‐Derived Structures

#### 3.3.1 Pyrene‐Conjugated 2PCA Tags Enable LALDI Measurements of Peptides

Label‐assisted laser desorption/ionization (LALDI)‐MS is a matrix‐free variant of MALDI‐MS in which matrix‐like molecules are coupled to analytes of interest by a covalent bond or chelation. For example, derivatization of carbohydrates with matrix‐like pyrene tags has enabled their LALDI‐based detection without the need for additional co‐crystallization with conventional matrices [45]. We aimed to evaluate whether 2PCA labeling of protein N‐termini can be combined with their (covalent) derivatization with a matrix‐like pyrene tag. We proposed that this approach would facilitate the selective ionization and detection of N‐terminal peptides in LALDI‐MS, without any biochemical enrichment as required for most LC‐ESI‐MS/MS‐based N‐terminomic strategies. We further hypothesized that LALDI‐based N‐terminomics would reduce the complexity of samples containing hundreds to thousands of proteoforms. Because mass spectrometry imaging (MSI) lacks chromatographic separation, this reduction in sample complexity may be crucial for establishing a mass spectrometry imaging (MSI)‐based N‐terminomic approach.

**FIGURE 3:**
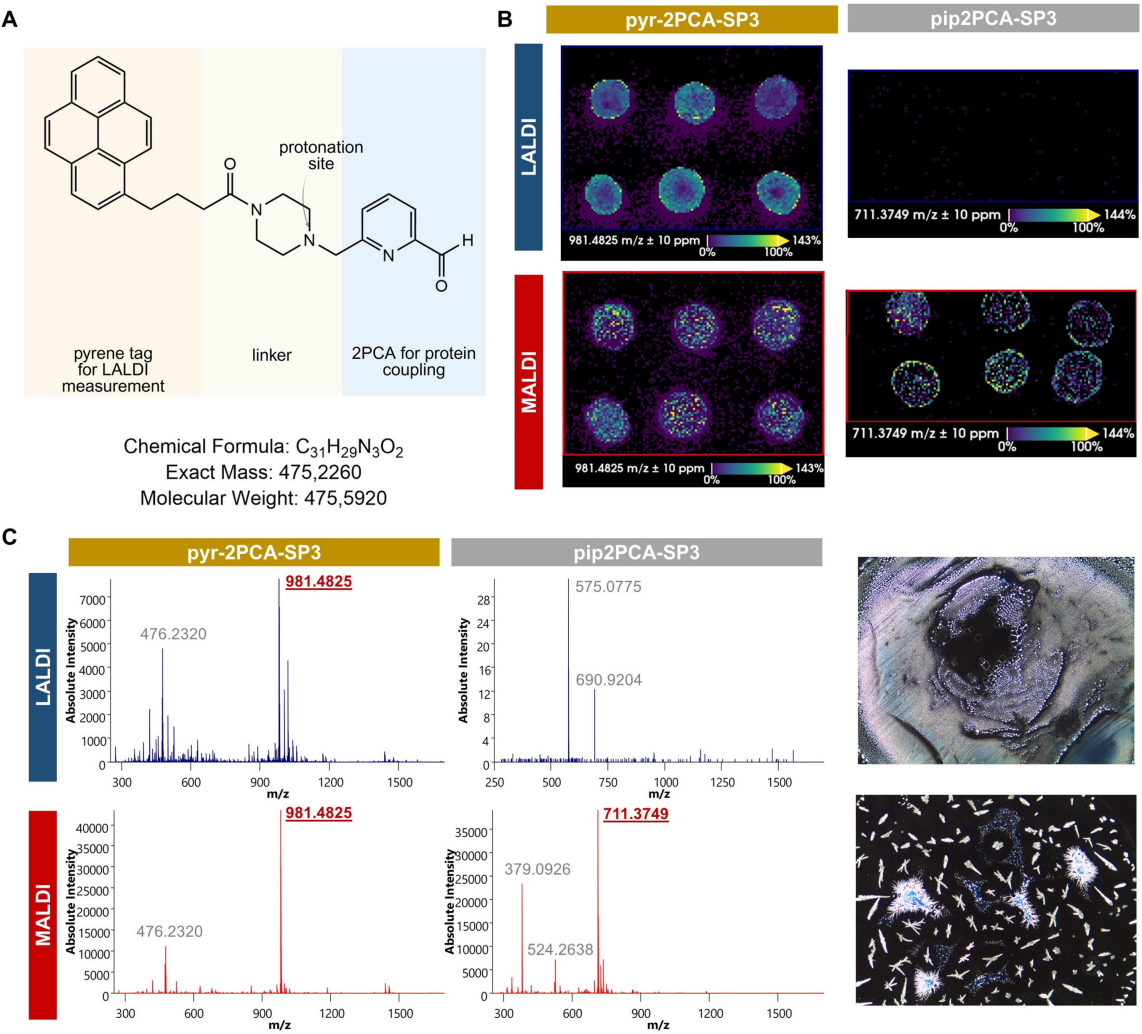
Pyr‐2PCA tags enable LALDI measurements. (A) Molecular structure of the pyr‐2PCA compound. (B) Ion images of the respective imaging runs are shown, corresponding to mean spectra visualized in C. (C) Mean spectra derived from a MS1 MALDI imaging run of six spots of SP3 (MRFA) labeled with pyr‐2PCA (left, *m*/*z* 981.4825) and pip2PCA (right, *m*/*z* 711.3749), respectively. First acquisition was carried out on spots without CHCA matrix (LALDI). Afterwards, the same spots were overspotted with CHCA matrix and remeasured (MALDI). The right section shows light microscopy images of 1 μL spots of pyr‐2PCA‐labeled SP3 and pip2PCA‐labeled SP3 mixed with CHCA matrix in a 1:1 ratio before spotting.

To evaluate these opportunities, we developed a novel labeling structure by conjugating the pip2PCA linker to a pyrene‐incorporating LALDI tag (pyr‐2PCA tag), thereby achieving selective coupling to N‐termini. The new pyr‐2PCA tag was synthesized via amide coupling between commercially available 1‐pyrenebutyric acid and a piperazine‐containing 2PCA derivative. The 2‐PCA intermediate was prepared in five steps starting from 2,6‐pyridinedimethanol according to the previously reported literature procedure [20]. The final amide bond formation proceeded smoothly under standard coupling conditions, yielding the desired pyr‐2PCA tag (Figure 3A). The entire synthesis route and NMR analysis of the pyr‐2PCA compound can be found in the Supporting Information (Figure S2).

For proof of concept, we tagged a synthetic peptide with the sequence MRFA (SP3) with either the pyr‐2PCA tag or the pyrene‐free pip2PCA tag. The pyr‐2PCA sequence was detected by LALDI whereas the solely pip2PCA‐tagged peptide remained undetectable in LALDI mode (Figure 3B,C). To account for variability in spot quality and laser shot performance, six replicate spots were imaged in LALDI mode. Overspotting the same spots with CHCA matrix enabled subsequent MALDI control measurements, which corroborated the LALDI‐based mass of the pyr‐2PCA‐tagged peptide and controlled for the presence of the pip2PCA‐tagged peptide (Figure 3B,C). We observed a different crystallization behavior and a different signal distribution within LALDI and MALDI spots. While CHCA‐mixed samples formed typical crystalline structures, the pyr‐2PCA‐labeled spots exhibited a film‐like morphology, consistent with the reported advantage of LALDI in minimizing matrix crystal size and improving homogeneity [29].

#### 3.3.2 Pyr‐2PCA Tags Inherit Co‐ and Self‐Ionization Effects

**FIGURE 4:**
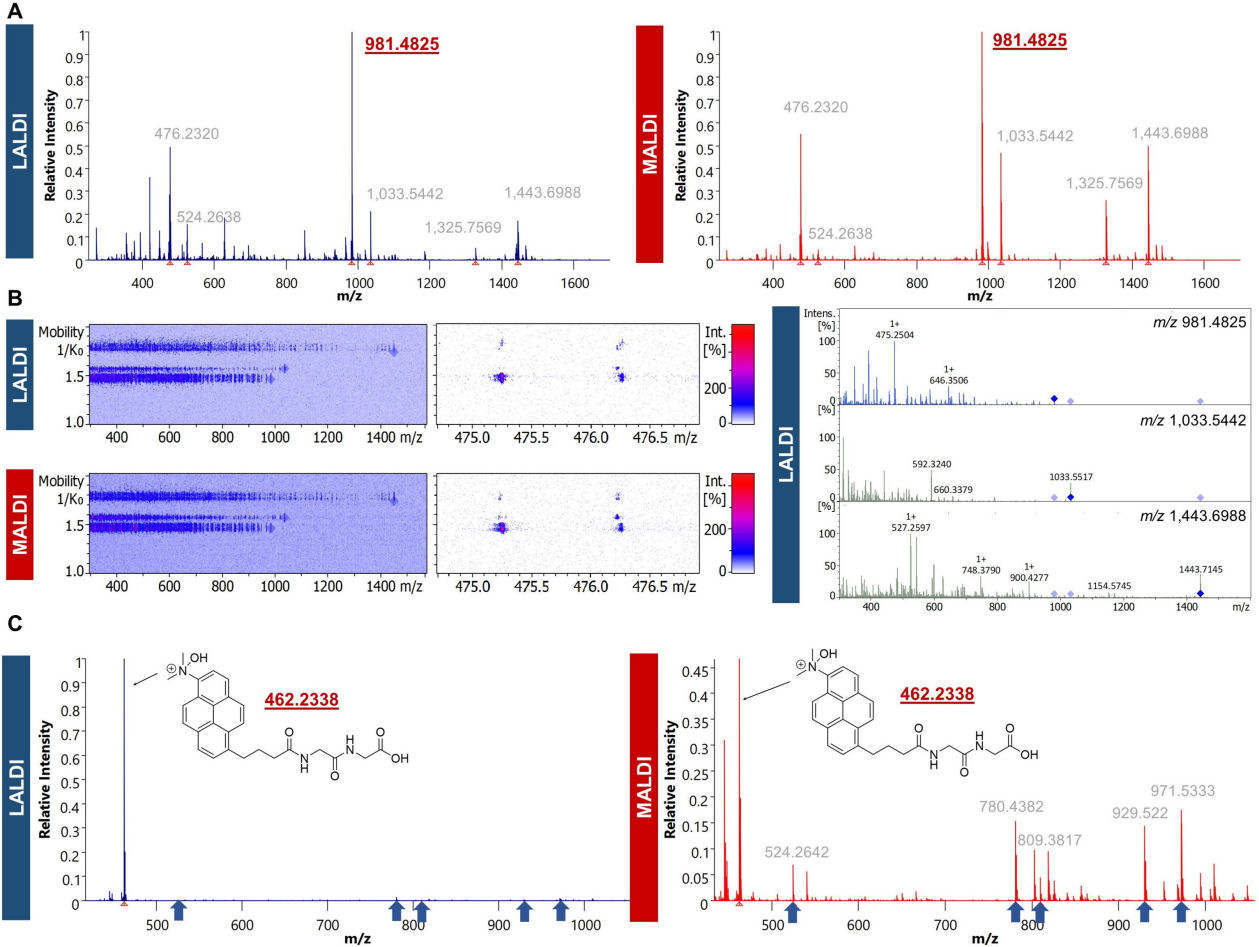
Co‐ionization effects by pyr‐2PCA. (A) Mean, RMS‐normalized mass spectra of MS1 imaging run of three spots containing pyr‐2PCA‐labeled SP3 (*m*/*z* 981.4825 (pyr‐2PCA‐MRFA)) including three spike‐in peptides (*m*/*z* 1033.5442 (LQAEAFQAR), *m*/*z* 1325.7569 (DNIQGITKPAIR), and *m*/*z* 1443.6988 (SP2)). LALDI and MALDI acquisition was performed according to Figure 3B. (B) iprm‐PASEF imaging measurement of two replicate spots of the same sample used in A, targeting three peptides including pyr‐2PCA‐SP3 at *m*/*z* 981.4825, *m*/*z* 1033.5442, and *m*/*z* 1443.6988. The left plot shows the *m*/*z*—mobility heatmap including a zoomed visualization of the reporter ion peak at *m*/*z* 475.2504, matching the pyr‐2PCA reporter ion fragment. The right plot shows the mean MS2 spectra for each trace that were extracted from the mobilogram (from top to bottom, MS2 spectra of *m*/*z* 981.4825, *m*/*z* 1033.5442, and *m*/*z* 1443.6988). (C) Mean, RMS‐normalized mass spectra of MS1 imaging run of one spot of a mixture of dma‐pyr‐GG (*m*/*z* 462.2338) with five synthetic spike‐in peptides (*m*/*z* 524.2642 (SP3), *m*/*z* 780.4382 (Ac‐AVRPGAPA‐OH), *m*/*z* 809.3817 (Ac‐AVAPGYPA‐OH+Na), *m*/*z* 929.522 (H‐AVRPGYP‐Lys (Ac)‐OH), and *m*/*z* 971.533 (Ac‐AVRPGYP‐Lys (Ac)‐OH)). LALDI and MALDI acquisition was performed as mentioned above.

A distinct co‐ionization effect was observed for pyr‐2PCA structures following solution‐phase labeling, with unlabeled spike‐in peptides remaining detectable by LALDI‐MS even in the absence of CHCA matrix co‐crystallization. Comparing LALDI‐MS and MALDI‐MS, a lower signal intensity of spike‐in peptides was observed in LALDI, even though the same spots were measured subsequently, following CHCA overspotting. Additionally, we observed a strong signal at *m*/*z* 476.2320, which corresponds to the fully intact pyr‐2PCA compound, suggesting that pyr‐2PCA was not completely removed during the C18‐based SPE cleanup and remained stable throughout the reaction incubation and subsequent processing steps. These observations suggest that the pyrene moiety of the LALDI tag has matrix‐like properties and is capable of promoting the desorption and ionization of co‐present unlabeled analytes, albeit with lower efficiency than standard matrices such as CHCA (Figure 4A). Further, we demonstrated that, even in LALDI mode, iprm‐PASEF measurements possess the capacity to distinguish between labeled and unlabeled specimens by reporter ion detection (Figure 4B).

The presence of unreacted pyr‐2PCA labeling reagent (*m*/*z* 476.232) highlights the requirement for its removal for potential applications in MSI. Washing steps in aqueous or organic solvents are commonly used in histological stainings that preserve morphological structures.

Yet, co‐ionization may also be promoted by protein‐reacted and coupled pyr‐2PCA. To assess this possibility, we synthesized a defined dipeptide mimetic dma‐pyr‐GG in which a dimethylamino‐pyrene group was conjugated to a Gly‐Gly scaffold, yielding a single molecular species (Figure S3). Upon LALDI MS1 analysis, we observed a mass increase of +17.02 Da, which we attribute to the spontaneous hydroxylation of the dimethylamino (–N (CH_3_)_2_) moiety. When analyzed in LALDI mode at varying concentrations and in mixtures with five unlabeled peptides, the pyrene‐conjugated dipeptide exhibited significantly enhanced detectability compared to unlabeled peptides under matrix‐free conditions (Figure 4C). The entire dilution series can be found in the Supporting Information (Figure S4). Importantly, the co‐ionization effect was concentration dependent, with signal detection of unlabeled species declining at lower concentrations of the pyrene compound. Therefore, these findings highlight the possibility of co‐ionization originating from LALDI tags while also supporting their potential for signal boosting of protein N‐termini in matrix‐free, LALDI analysis.

#### 3.3.3 N‐Terminal Selectivity in LALDI by Pyr‐2PCA Labeling

**FIGURE 5:**
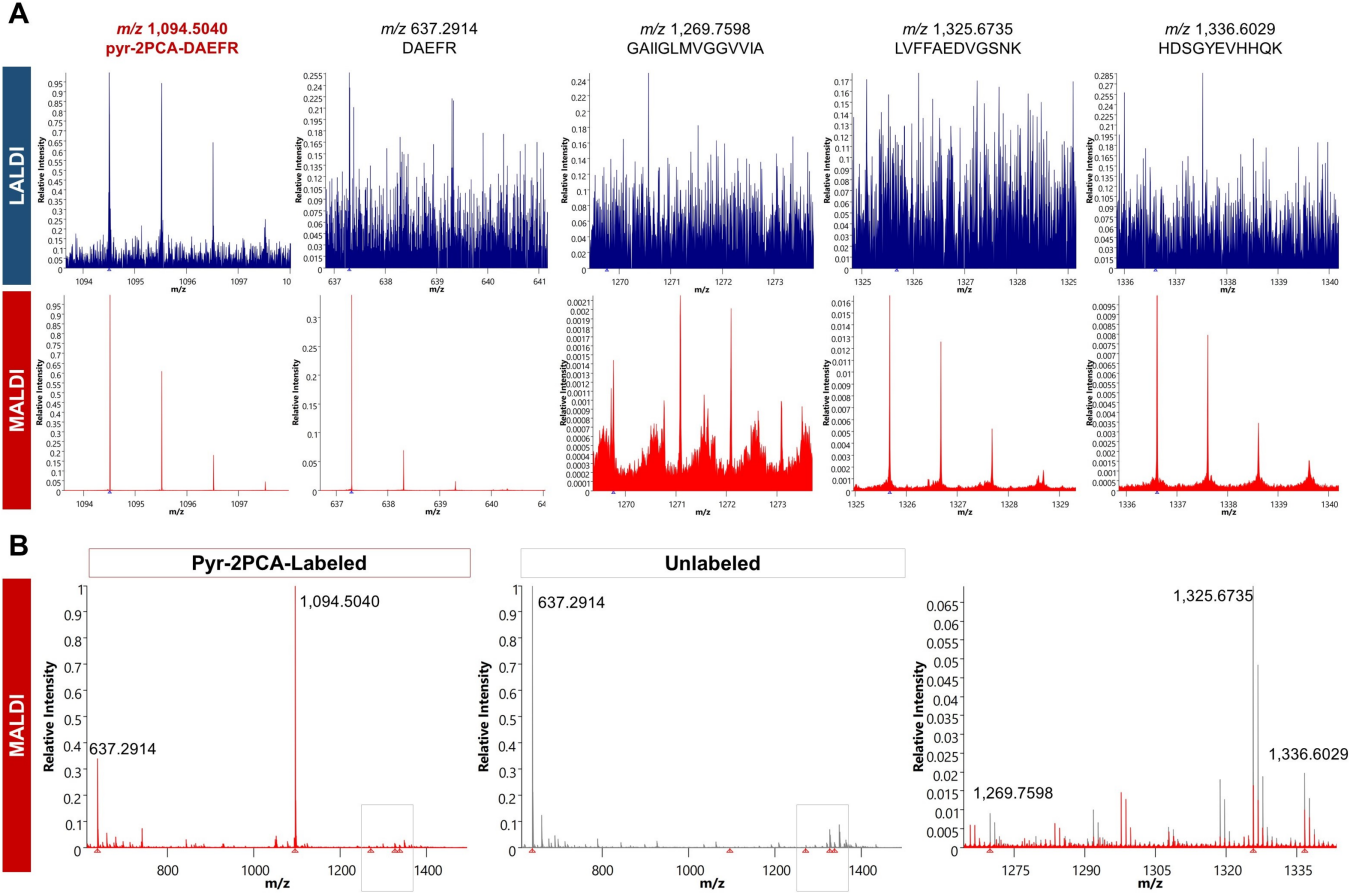
Pyr‐2PCA labeling of β‐amyloid before tryptic digestion. (A) Mean, RMS‐normalized spectra of an imaging run of three spots of pyr‐2PCA‐labeled β‐amyloid (DAEFRHDSGYEVHHQKLVFFAEDVGSNKGAIIGLMVGGVVIA), tryptic digested, in LALDI and MALDI mode. Spectra are zoomed on the respective tryptic peptides of β‐amyloid. The labeled, N‐terminal peptide (pyr‐2PCA‐DAEFR, *m*/*z* 1094.5040) is the only *m*/*z* with a clean signal in LALDI mode. (B) Mean, RMS‐normalized spectra of an imaging run of three spots of pyr‐2PCA‐labeled (red) and unlabeled (grey) β‐amyloid, tryptic digested. The right spectrum shows an overlay of both mean spectra, zoomed into the mass range of the three tryptic peptides HDSGYEVHHQK (*m*/*z* 1336.6029), LVFFAEDVGSNK (*m*/*z* 1325.6735), and GAIIGLMVGGVVIA (*m*/*z* 1269.7598), indicating a higher intensity of the not N‐terminal peptides in the control compared to the labeled sample.

We aimed to characterize the usage of pyr‐2PCA labeling on the protein level for selective N‐terminus detection. For this purpose, β‐amyloid was utilized as a synthetic test peptide containing three tryptic cleavage sites, as described in Section 3.2.1. After in‐solution labeling with pyr‐2PCA, C18‐based SPE, and tryptic digestion, LALDI‐MS1 analysis revealed selective detection of the labeled N‐terminal peptide DAEFR. Here, the mean spectrum obtained from an imaging run of three replicate and matrix‐free spots exhibited a dominant signal at *m*/*z* 1094.5040, corresponding to the expected mass of the pyr‐2PCA‐modified N‐terminal tryptic peptide of β‐amyloid (pyr‐2PCA‐DAEFR). The other three generated tryptic peptides from β‐amyloid remained undetectable in LALDI, whereas the unlabeled N‐terminal peptide could be detected with a very low intensity and always alongside the labeled species (Figure 5A).

Additionally, we observed a change in signal intensity ratios when comparing the pyr‐2PCA‐labeled sample with the unlabeled control digest in MALDI mode: The relative abundance of the pyr‐2PCA‐labeled N‐terminal peptide to tryptic peptides increased compared to the ratio observed for the unlabeled N‐terminal species in the control measurement (Figure 5B). This finding supports the signal‐enhancing effect of the pyr‐2PCA tag, even when analyzed under conventional CHCA‐assisted MALDI conditions.

Therefore, these results confirm that pyr‐2PCA labeling enhances the detectability of N‐terminal peptides when applied prior to tryptic digestion. Furthermore, no significant off‐target labeling of lysine ε‐amines was detected, indicating high selectivity of the 2PCA‐labeling strategy.

## 4 Discussion

In this study, we present pyrene‐conjugated 2PCA tags (pyr‐2PCA) that enable a matrix‐free and selective detection of N‐termini. Therefore, this is the first step to set up a tool for studying proteolytic processes on a spatial level in a high‐throughput and antibody‐independent manner. Here, we characterized 2PCA labeling by MALDI‐MS and investigated the use of pyr‐2PCA tags for LALDI‐MS and signal boosting in MALDI‐MS. We were able to show selective labeling by 2PCA upon aqueous removal of excess labeling reagent, detection of a reporter ion in MALDI MS/MS mode, and N‐terminal selectivity in LALDI using pyr‐2PCA.

In a broad perspective, these tags target several limitations of MALDI imaging of tryptic peptides that evolve from the lack of chromatographic separation, particularly spectral crowding and TIMS cell oversampling by matrix ions. By selectively boosting a subset of peptides and overcoming the need for additional matrix deposition, pyrene‐coupled tags hold the potential to expand the accessible set of tryptic peptides and possibly target specific peptides of interest in LALDI/MALDI imaging.

As this report provides a preliminary proof of concept, there are several aspects that require further optimization in order to make this tool applicable for biological investigations. First, there is a necessity to evaluate and potentially enhance the efficiency of in situ labeling. In order to achieve this, different antigen retrieval buffers can be evaluated, the incorporation of detergents during the labeling step can be tested to improve tissue permeability and target accessibility, and repeated cycles of labeling reagent incubation can be applied [46]. Second, the detection sensitivity of the LALDI tags can be enhanced, for instance, by structural modifications of the pyrene moiety, such as substitution by a N,N‐dimethylamino group, as reported previously [47]. At last, MS acquisition methods can be further refined by optimizing tuning parameters relevant for TIMS cell ion storage, which could improve accumulation efficiency and therefore sensitivity in a targeted manner. Possibly, acquisition methods can be designed to focus on a specific *m*/*z* and 1/*K*_0_ window that contains only a single, distinct signal.

Several potential applications emerge for the principle of 2PCA‐supported labeling in the field of imaging of tryptic peptides, the most straightforward being the detection and identification of neo‐N‐termini by mass shift on MS1 level and possible signal selectivity by pyrene incorporation. However, the 2PCA linker offers versatility beyond pyrene‐tag incorporation as it can be conjugated with other functional groups such as aromatic systems that significantly increase the 1/*K*_0_ value of the attached compound and therefore improve separation by trapped ion mobility. Additionally, metal‐based structures can be introduced to the tag, enabling clean distinction from unlabeled signals on MS1 level via distinct isotope patterns. Further, these N‐terminal tags can help with integration and alignment between LC–MS/MS and MALDI imaging data, as the mass shift detection can serve as an additional parameter for matching, thereby improving the reliability of obtained peptide identifications.

In summary, this study introduces a novel and foundational approach employing pyrene‐conjugated 2PCA tags for the selective labeling of protein N‐termini, enabling matrix‐free and signal‐enhanced detection in MALDI‐ and LALDI‐MS. This strategy represents an initial step toward boosting tryptic peptide signals in MALDI, analogous to the established concept of on‐tissue chemical derivatization in metabolite imaging for targeted analyte detection [48]. While the present study serves as a proof of concept, further optimization will be essential to fully realize the potential of this platform. Ultimately, this approach offers a promising route for high‐throughput, antibody‐independent investigation of proteolytic processes with spatial resolution.

## Author Contributions

**Mujia Jenny Li:** conceptualization, methodology, data curation, writing – original draft, formal analysis. **Murat Kucukdisli:** conceptualization, chemical compound synthesis. **Florian Braun:** conceptualization, chemical compound synthesis. **David W. Will:** conceptualization. **Nadine Meier:** data curation. **Bettina Wehrle:** data curation. **Larissa Chiara Meyer:** methodology, writing – review and editing. **Melanie Christine Föll:** funding acquisition, writing – review and editing. **Oliver Schilling:** conceptualization, project administration, funding acquisition, supervision, writing – review and editing.

## Funding

Prof. Dr. Oliver Schilling acknowledges funding by the Deutsche Forschungsgemeinschaft (DFG, projects 446058856, 466359513, 444936968, 405351425, and 431336276), 43198400 (SFB 1453 “NephGen”), 441891347 (SFB 1479 “OncoEscape”), 423813989 (GRK 2606 “ProtPath”), 322977937 (GRK 2344 “MeInBio”), the ERA PerMed program (BMBF, 01KU1916 and 01KU1915A), the ERA TransCan program (BMBF 01KT2201,“PREDICO”; 01KT2333, “ICC‐STRAT”), the German Consortium for Translational Cancer Research (project Impro‐Rec), the investBW program (project BW1_1198/03 “KASPAR”), the BMBF KMUi program (project 13GW0603E and project ESTHER), and BMBF FKZ 03ZU1208AA (nanodiag BW).

Open access funding enabled and organized by Projekt DEAL.

## Supporting information


**Table S1:** Overview of all used peptides and compounds, (Ac) = N‐acetylation, [16] = hydroxylation.

**Figure S1:** MS/MS spectra with highlighted b‐ and y‐ions of (A) SP1 unlabeled and (B) DNIQGITKPAIR labeled with 2PCA. Plots were generated in Python using the pyteomics package. Intensity thresholds were set to (A) 200 and (B) 40. Matching with theoretical masses was performed with a tolerance of 0.3 Da. For annotation of the sequence (top right in the individual spectrum), the respective theoretical fragment ion m/z values are printed. (C) Zoomed MS1 spectrum (corresponding to Figure 1B SP1 + pip2PCA reaction) showing the peaks corresponding to double labeled SP1 with a low intensity after aqueous dilution.
**Figure S2:** Pyr‐2PCA synthesis and NMR analysis. (A) Synthesis of pyr‐2PCA. (B) Analysis of pyr‐2PCA, including MS1 spectrum and 1H NMR (400 MHz, DMSO‐d6) spectrum.
**Figure S3:** Dma‐Pyr‐GG synthesis and analysis by NMR and MS. (A) Synthesis route for dma‐pyr‐GG. (B) MS1 analysis on TimsTOF Flex after short‐term storage, exhibiting a mass shift of 16 Da. (C) MS1 mass spectrum directly after synthesis and 1H NMR (400 MHz, CD3OD) spectrum of dma‐pyr‐GG.
**Figure S4:** Mean, RMS‐normalized spectra of one spot measured in LALDI or MALDI imaging mode, respectively, of dma‐pyr‐GG mixed with different ratios to five spike‐in peptides (see Section 2.6).

## Data Availability

The data that support the findings of this study are available from the corresponding author upon reasonable request.

## References

[1] O. Schilling and P. Findeisen , “Proteases and Disease,” Proteomics. Clinical Applications 8, no. 5–6 (2014): 296–298, 10.1002/prca.201470035. 24913842

[2] J. S. Bond , “Proteases: History, Discovery, and Roles in Health and Disease,” Journal of Biological Chemistry 294, no. 5 (2019): 1643–1651, 10.1074/jbc.TM118.004156. 30710012 PMC6364759

[3] M. Schuliga , “The Inflammatory Actions of Coagulant and Fibrinolytic Proteases in Disease,” Mediators of Inflammation 2015 (2015): 437695, 10.1155/2015/437695. 25878399 PMC4387953

[4] C. C. Taggart , C. M. Greene , T. P. Carroll , S. J. O'Neill , and N. G. McElvaney , “Elastolytic Proteases: Inflammation Resolution and Dysregulation in Chronic Infective Lung Disease,” American Journal of Respiratory and Critical Care Medicine 171, no. 10 (2005): 1070–1076, 10.1164/rccm.200407-881PP. 15695494

[5] C. Bonnans , J. Chou , and Z. Werb , “Remodelling the Extracellular Matrix in Development and Disease,” Nature Reviews. Molecular Cell Biology 15, no. 12 (2014): 786–801, 10.1038/nrm3904. 25415508 PMC4316204

[6] A. M. Haack , C. M. Overall , and U. Auf dem Keller , “Degradomics Technologies in Matrisome Exploration,” Matrix Biology 114 (2022): 1–17, 10.1016/j.matbio.2022.10.003. 36280126

[7] J. S. Lewis, 2nd , C. M. Terriff , D. R. Coulston , and M. W. Garrison , “Protease Inhibitors: A Therapeutic Breakthrough for the Treatment of Patients With Human Immunodeficiency Virus,” Clinical Therapeutics 19, no. 2 (1997): 187–214, 10.1016/s0149-2918(97)80110-5. 9152561

[8] A. Eatemadi , H. T. Aiyelabegan , B. Negahdari , et al., “Role of Protease and Protease Inhibitors in Cancer Pathogenesis and Treatment,” Biomedicine and Pharmacotherapy 86 (2017): 221–231, 10.1016/j.biopha.2016.12.021. 28006747

[9] J. Dorward , O. Gbinigie , T. Cai , et al., “The Protease Inhibitor Lopinavir, Boosted With Ritonavir, as Treatment for COVID‐19: A Rapid Review,” Antiviral Therapy 25, no. 7 (2020): 365–376, 10.3851/imp3385. 33704086

[10] J. M. Pawlotsky , “The Science of Direct‐Acting Antiviral and Host‐Targeted Agent Therapy,” Antiviral Therapy 17, no. 6 Pt B (2012): 1109–1117, 10.3851/imp2423. 23188746

[11] N. Holgersen , V. W. Nielsen , N. A. L. Rosenø , et al., “Biomarkers of Systemic Inflammation Are Associated With Disease Severity and Metabolic Syndrome in Patients With Hidradenitis Suppurativa,” JAAD International 15 (2024): 170–178, 10.1016/j.jdin.2024.03.002. 38638915 PMC11025002

[12] K. Kalogeropoulos , L. Bundgaard , and U. Auf dem Keller , “Sensitive and High‐Throughput Exploration of Protein N‐Termini by TMT‐TAILS N‐Terminomics,” Methods in Molecular Biology 2718 (2023): 111–135, 10.1007/978-1-0716-3457-8_7. 37665457

[13] A. Derakhshani , M. Bulluss , R. Penner , and A. Dufour , “N‐Terminomics/TAILS of Human Tumor Biopsies and Cancer Cell Lines,” Methods in Molecular Biology 2747 (2024): 19–28, 10.1007/978-1-0716-3589-6_2. 38038928

[14] M. Mintoo , A. Chakravarty , and R. Tilvawala , “N‐Terminomics Strategies for Protease Substrates Profiling,” Molecules 26, no. 15 (2021): 4699, 10.3390/molecules26154699. 34361849 PMC8348681

[15] M. Cosenza‐Contreras , A. Seredynska , D. Vogele , et al., “TermineR: Extracting Information on Endogenous Proteolytic Processing From Shotgun Proteomics Data,” Proteomics 24, no. 19 (2024): e2300491, 10.1002/pmic.202300491. 39126236

[16] H. Shahinian , S. Tholen , and O. Schilling , “Proteomic Identification of Protease Cleavage Sites: Cell‐Biological and Biomedical Applications,” Expert Review of Proteomics 10, no. 5 (2013): 421–433, 10.1586/14789450.2013.841547. 24117201

[17] D. Bertaccini , S. Vaca , C. Carapito , F. Arsène‐Ploetze , A. Van Dorsselaer , and C. Schaeffer‐Reiss , “An Improved Stable Isotope N‐Terminal Labeling Approach With Light/Heavy TMPP to Automate Proteogenomics Data Validation: dN‐TOP,” Journal of Proteome Research 12, no. 6 (2013): 3063–3070, 10.1021/pr4002993. 23641718

[18] M. Baudet , P. Ortet , J.‐C. Gaillard , et al., “Proteomics‐Based Refinement of Deinococcus Deserti Genome Annotation Reveals an Unwonted Use of Non‐Canonical Translation Initiation Codons,” Molecular and Cellular Proteomics 9, no. 2 (2010): 415–426, 10.1074/mcp.M900359-MCP200. 19875382 PMC2830850

[19] B. Fernandez , J. Armengaud , G. Subra , and C. Enjalbal , “MALDI‐MS/MS of N‐Terminal TMPP‐Acyl Peptides: A Worthwhile Tool to Decipher Protein N‐Termini,” European Journal of Organic Chemistry 2022, no. 21 (2022): e202101549, 10.1002/ejoc.202101549.

[20] J. I. MacDonald , H. K. Munch , T. Moore , and M. B. Francis , “One‐Step Site‐Specific Modification of Native Proteins With 2‐Pyridinecarboxyaldehydes,” Nature Chemical Biology 11, no. 5 (2015): 326–331, 10.1038/nchembio.1792. 25822913

[21] C. Wolf , A. Behrens , C. Brungs , et al., “Mobility‐Resolved Broadband Dissociation and Parallel Reaction Monitoring for Laser Desorption/Ionization‐Mass Spectrometry – Tattoo Pigment Identification Supported by Trapped ion Mobility Spectrometry,” Analytica Chimica Acta 1242 (2023): 340796, 10.1016/j.aca.2023.340796. 36657890

[22] S. Heuckeroth , A. Behrens , C. Wolf , et al., “On‐Tissue Dataset‐Dependent MALDI‐TIMS‐MS2 Bioimaging,” Nature Communications 14, no. 1 (2023): 7495, 10.1038/s41467-023-43298-9. PMC10657435 37980348

[23] K. V. Djambazova , M. Dufresne , L. G. Migas , et al., “MALDI TIMS IMS of Disialoganglioside Isomers─GD1a and GD1b in Murine Brain Tissue,” Analytical Chemistry 95, no. 2 (2023): 1176–1183, 10.1021/acs.analchem.2c03939. 36574465

[24] E. Rudt , M. Froning , S. Heuckeroth , et al., “Rapid MALDI‐MS/MS‐Based Profiling of Lipid A Species From Gram‐Negative Bacteria Utilizing Trapped Ion Mobility Spectrometry and mzmine,” Analytical Chemistry 97, no. 14 (2025): 7781–7788, 10.1021/acs.analchem.4c05989. 40167996 PMC12004357

[25] J. Li Mujia , C. Meyer Larissa , N. Meier , et al., “Spatial Proteomics by Parallel Accumulation‐Serial Fragmentation Supported MALDI MS/MS Imaging: A First Glance Into Multiplexed and Spatial Peptide Identification,” Rapid Communications in Mass Spectrometry 39, no. 9 (2025): e10006, 10.1002/rcm.10006. 39910729 PMC11799399

[26] T. D. McCarley , R. L. McCarley , and P. A. Limbach , “Electron‐Transfer Ionization in Matrix‐Assisted Laser Desorption/Ionization Mass Spectrometry,” Analytical Chemistry 70, no. 20 (1998): 4376–4379, 10.1021/ac980527i.

[27] J. W. Szewczyk , R. L. Zuckerman , R. G. Bergman , and J. A. Ellman , “A Mass Spectrometric Labeling Strategy for High‐Throughput Reaction Evaluation and Optimization: Exploring C‐H Activation,” Angewandte Chemie (International Ed. in English) 40, no. 1 (2001): 216–219, 10.1002/1521-3773(20010105)40:1<216::Aid-anie216>3.0.Co;2-k. 29711936

[28] J. R. Cabrera‐Pardo , D. I. Chai , S. Liu , M. Mrksich , and S. A. Kozmin , “Label‐Assisted Mass Spectrometry for the Acceleration of Reaction Discovery and Optimization,” Nature Chemistry 5, no. 5 (2013): 423–427, 10.1038/nchem.1612. PMC4512672 23609094

[29] A. Mandal , M. Singha , P. S. Addy , and A. Basak , “Laser Desorption Ionization Mass Spectrometry: Recent Progress in Matrix‐Free and Label‐Assisted Techniques,” Mass Spectrometry Reviews 38, no. 1 (2019): 3–21, 10.1002/mas.21545. 29029360

[30] P. S. Addy , S. Basu Roy , S. M. Mandal , and A. Basak , “Polyaromatic Label‐Assisted Laser Desorption Ionization Mass Spectrometry (LA‐LDI MS): A New Analytical Technique for Selective Detection of Zinc Ion,” RSC Advances 4, no. 44 (2014): 23314–23318, 10.1039/C4RA02250E.

[31] P. S. Addy , A. Bhattacharya , S. M. Mandal , and A. Basak , “Label‐Assisted Laser Desorption/Ionization Mass Spectrometry (LA‐LDI‐MS): An Emerging Technique for Rapid Detection of Ubiquitous *cis*‐1,2‐Diol Functionality,” RSC Advances 4, no. 87 (2014): 46555–46560, 10.1039/C4RA07499H.

[32] K. Yoneda , Y. Hu , R. Watanabe , M. Kita , and H. Kigoshi , “Binding Position Analysis of Target Proteins With the Use of Amidopyrene Probes as LA‐LDI Enhancing Tags,” Organic and Biomolecular Chemistry 14, no. 36 (2016): 8564–8569, 10.1039/c6ob01381c. 27545719

[33] V. Broeckx , L. Peeters , E. Maes , L. Pringels , E. T. Verjans , and B. Landuyt , “Formalin‐Fixed Paraffin‐Embedded Tissue: The Holy Grail of Clinical Proteomics,” Proteomics. Clinical Applications 8, no. 9–10 (2014): 735–736, 10.1002/prca.201400132. 25231873

[34] L. I. Levitsky , J. A. Klein , M. V. Ivanov , and M. V. Gorshkov , “Pyteomics 4.0: Five Years of Development of a Python Proteomics Framework,” Journal of Proteome Research 18, no. 2 (2019): 709–714, 10.1021/acs.jproteome.8b00717. 30576148

[35] H. N. Bridge , W. Leiter , C. L. Frazier , and A. M. Weeks , “An N Terminomics Toolbox Combining 2‐Pyridinecarboxaldehyde Probes and Click Chemistry for Profiling Protease Specificity,” Cell Chemical Biology 31, no. 3 (2024): 534–49.e8, 10.1016/j.chembiol.2023.09.009. 37816350 PMC10960722

[36] H. N. Bridge and A. M. Weeks , “Proteome‐Derived Peptide Libraries for Deep Specificity Profiling of N‐Terminal Modification Reagents,” Current Protocols 3, no. 6 (2023): e798, 10.1002/cpz1.798. 37283519 PMC10338020

[37] L. Zhai , L. Wang , H. Hu , et al., “PBC, an Easy and Efficient Strategy for High‐Throughput Protein C‐Terminome Profiling,” Frontiers in Cell and Developmental Biology 10 (2022): 995590, 10.3389/fcell.2022.995590. 36120566 PMC9471192

[38] H. Zhang , S. Chacko , and J. R. Cannon , “2‐Pyridine Carboxaldehyde for Semi‐Automated Soft Spot Identification in Cyclic Peptides,” International Journal of Molecular Sciences 23, no. 8 (2022): 4269, 10.3390/ijms23084269. 35457087 PMC9028278

[39] R. Krüger and M. Karas , “Formation and Fate of Ion Pairs During MALDI Analysis: Anion Adduct Generation as an Indicative Tool to Determine Ionization Processes,” Journal of the American Society for Mass Spectrometry 13, no. 10 (2002): 1218–1226, 10.1016/S1044-0305(02)00450-6. 12387328

[40] M. Karas and R. Krüger , “Ion Formation in MALDI: The Cluster Ionization Mechanism,” Chemical Reviews 103, no. 2 (2003): 427–440, 10.1021/cr010376a. 12580637

[41] J. F. Leite , M. R. Hajivandi , T. Diller , and R. M. Pope , “Removal of Sodium and Potassium Adducts Using a Matrix Additive During Matrix‐Associated Laser Desorption/Ionization Time‐Of‐Flight Mass Spectrometric Analysis of Peptides,” Rapid Communications in Mass Spectrometry 18, no. 23 (2004): 2953–2959, 10.1002/rcm.1711. 15536629

[42] S. Trimpin , E. D. Inutan , T. N. Herath , and C. N. McEwen , “Matrix‐Assisted Laser Desorption/Ionization Mass Spectrometry Method for Selectively Producing Either Singly or Multiply Charged Molecular Ions,” Analytical Chemistry 82, no. 1 (2010): 11–15, 10.1021/ac902066s. 19904915

[43] Y. Li and R. B. Cole , “Charge State Distributions in Electrospray and MALDI” in Electrospray and MALDI Mass Spectrometry: Fundamentals, Instrumentation, Practicalities, and Biological Applications, 2nd ed., ed. R. B. Cole (John Wiley & Sons, 2010), 491–534.

[44] T. Tayri‐Wilk , M. Slavin , J. Zamel , et al., “Mass Spectrometry Reveals the Chemistry of Formaldehyde Cross‐Linking in Structured Proteins,” Nature Communications 11, no. 1 (2020): 3128, 10.1038/s41467-020-16935-w. PMC7305180 32561732

[45] J. R. Hauser , E. T. Bergström , A. N. Kulak , S. L. Warriner , J. Thomas‐Oates , and R. S. Bon , “Pyrene Tags for the Detection of Carbohydrates by Label‐Assisted Laser Desorption/Ionisation Mass Spectrometry,” Chembiochem 22, no. 8 (2021): 1430–1439, 10.1002/cbic.202000721. 33296552

[46] Y. Xu , T. M. Lih , A. M. De Marzo , Q. K. Li , and H. Zhang , “SPOT: Spatial Proteomics Through On‐Site Tissue‐Protein‐Labeling,” Clinical Proteomics 21, no. 1 (2024): 60, 10.1186/s12014-024-09505-5. 39443867 PMC11515502

[47] A. Arai , R. Watanabe , A. Hattori , et al., “N,N‐Dimethylaminopyrene as a Fluorescent Affinity Mass Tag for Ligand‐Binding Mode Analysis,” Scientific Reports 10, no. 1 (2020): 7311, 10.1038/s41598-020-64321-9. 32355254 PMC7192892

[48] Q. Zhou , A. Fülöp , and C. Hopf , “Recent Developments of Novel Matrices and On‐Tissue Chemical Derivatization Reagents for MALDI‐MSI,” Analytical and Bioanalytical Chemistry 413, no. 10 (2021): 2599–2617, 10.1007/s00216-020-03023-7. 33215311 PMC8007514

