# Supplementary material for: Pyrene‐Conjugated, 2‐Pyridinecarboxaldehyde Derivatives as N‐Terminus‐Specific Tags for MALDI‐ and LALDI‐MS
Source: Rapid Commun Mass Spectrom. 2026 Jan 20;40(8):e70034. doi: 10.1002/rcm.70034 (PMC12817326; doi:10.1002/rcm.70034)
Supplement: Supplementary file 1 — Table S1: Overview of all used peptides and compounds, (Ac) = N‐acetylation, [16] = hydroxylation. [file RCM-40-e70034-s002.docx]

**Table S1:** Overview of all used peptides and compounds, (Ac) = N-acetylation, [16] = hydroxylation.

| **sequence/molecule structure** | **mass** | **vendor** | **abbreviation (optional)** |
| --- | --- | --- | --- |
| AVRPGYPK | 886.492 | Intavis Peptide Services GmbH | SP1 |
| GSAGPPGATGFP[16]GAAGR | 1,442.685 | Intavis Peptide Services GmbH | SP2 |
| MRFA | 523.247 | Sigma Aldrich/Merck KGaA | SP3 |
| DAEFRHDSGYEVHHQKLVFFAEDVGSNKGAIIGLMVGGVVIA | 4,511.259 | AnaSpec | β-amyloid |
| AVRPGYPK(Ac) | 928.502 | Intavis Peptide Services GmbH |  |
| A(Ac)VRPGYPK | 928.502 | Intavis Peptide Services GmbH |  |
| A(Ac)VRPGYPK(Ac) | 970.520 | Intavis Peptide Services GmbH |  |
| DNIQGITKPAIR | 1,324.735 | Intavis Peptide Services GmbH |  |
| LQAEAFQAR | 1,032.524 | Intavis Peptide Services GmbH |  |
| A(Ac)VRPGAPA | 779.418 | Intavis Peptide Services GmbH |  |
| A(Ac)VAPGYPA | 786.380 | Intavis Peptide Services GmbH |  |
| DGDLCGAPYPAVRAAGPKTPIVSGR | 2,467.253 + 56.08 (tert-butyl) | Prof. Henning Jessen |  |
| 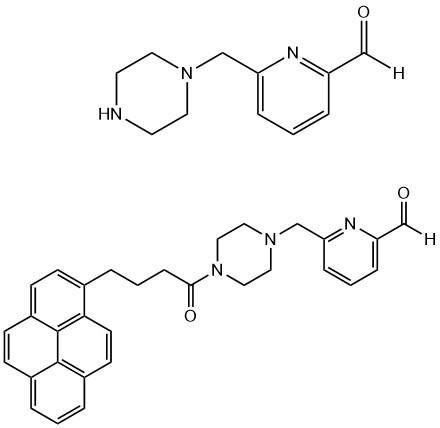 | 205.122 | Synthesis by EMBL | Pip2PCA |
| 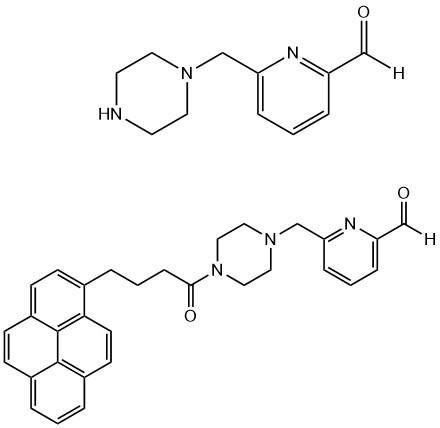 | 475.226 | Synthesis by EMBL | Pyr-2PCA |
| 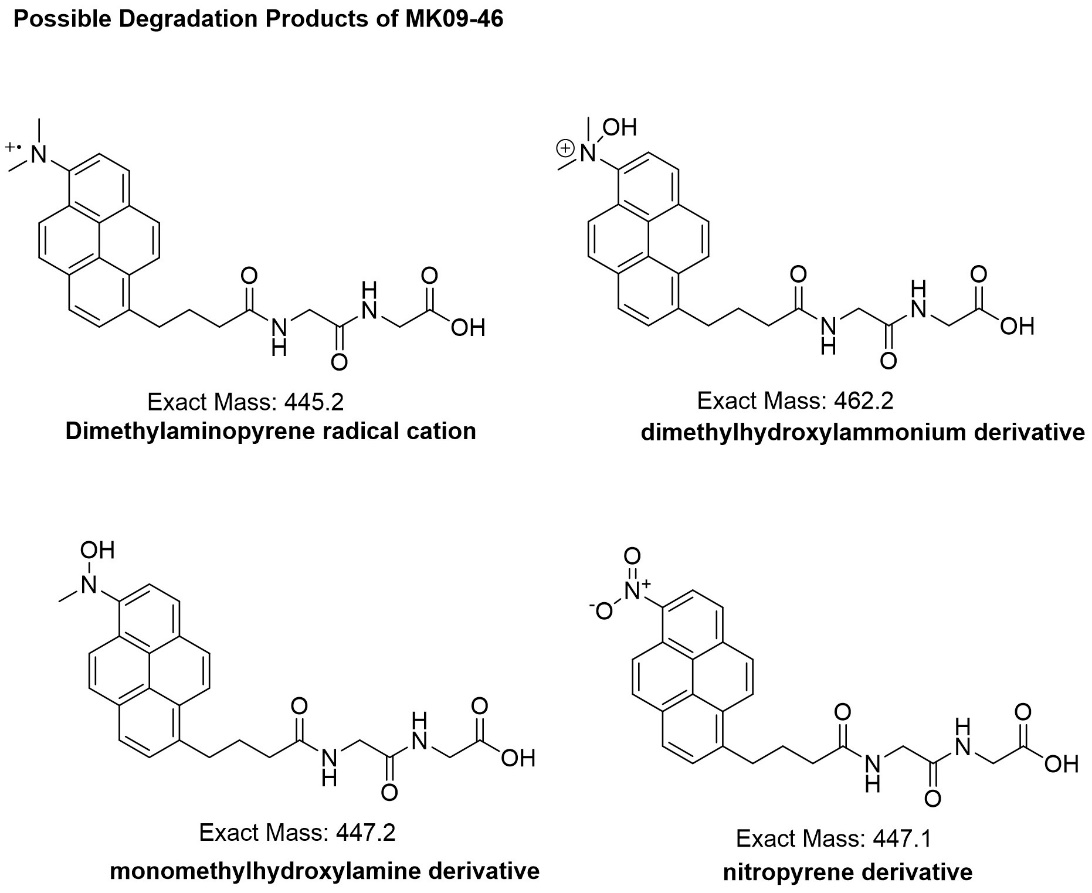 | 462.234 | Synthesis by EMBL | dma-pyr-GG |
