# Supplementary material for: Pyrene‐Conjugated, 2‐Pyridinecarboxaldehyde Derivatives as N‐Terminus‐Specific Tags for MALDI‐ and LALDI‐MS
Source: Rapid Commun Mass Spectrom. 2026 Jan 20;40(8):e70034. doi: 10.1002/rcm.70034 (PMC12817326; doi:10.1002/rcm.70034)
Supplement: Supplementary file 2 — Figure S1: MS/MS spectra with highlighted b‐ and y‐ions of (A) SP1 unlabeled and (B) DNIQGITKPAIR labeled with 2PCA. Plots were generated in Python using the pyteomics package. Intensity thresholds were set to (A) 200 and (B) 40. Matching with theoretical masses was performed with a tolerance of 0.3 Da. For annotation of the sequence (top right in the individual spectrum), the respective theoretical fragment ion m/z values are printed. (C) Zoomed MS1 spectrum (corresponding to Figure 1B SP1 + pip2PCA reaction) showing the peaks corresponding to double labeled SP1 with a low intensity after aqueous dilution. Figure S2: Pyr‐2PCA synthesis and NMR analysis. (A) Synthesis of pyr‐2PCA. (B) Analysis of pyr‐2PCA, including MS1 spectrum and 1H NMR (400 MHz, DMSO‐d6) spectrum. Figure S3: Dma‐Pyr‐GG synthesis and analysis by NMR and MS. (A) Synthesis route for dma‐pyr‐GG. (B) MS1 analysis on TimsTOF Flex after short‐term storage, exhibiting a mass shift of 16 Da. (C) MS1 mass spectrum directly after synthesis and 1H NMR (400 MHz, CD3OD) spectrum of dma‐pyr‐GG. Figure S4: Mean, RMS‐normalized spectra of one spot measured in LALDI or MALDI imaging mode, respectively, of dma‐pyr‐GG mixed with different ratios to five spike‐in peptides (see Section 2.6). [file RCM-40-e70034-s001.pptx]

## Slide 1
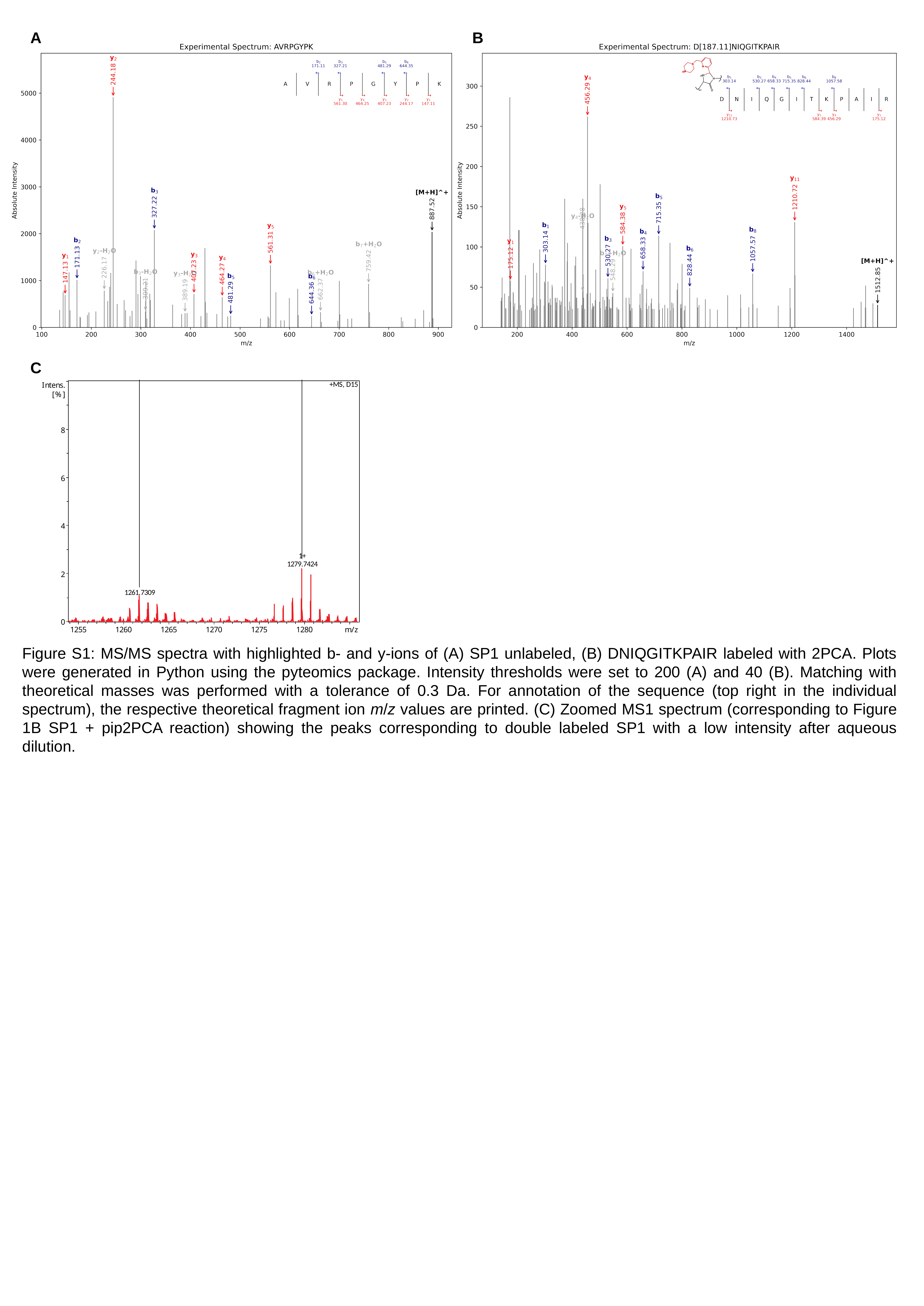

B
A
C
Figure S1: MS/MS spectra with highlighted b- and y-ions of (A) SP1 unlabeled, (B) DNIQGITKPAIR labeled with 2PCA. Plots were generated in Python using the pyteomics package. Intensity thresholds were set to 200 (A) and 40 (B). Matching with theoretical masses was performed with a tolerance of 0.3 Da. For annotation of the sequence (top right in the individual spectrum), the respective theoretical fragment ion m/z values are printed. (C) Zoomed MS1 spectrum (corresponding to Figure 1B SP1 + pip2PCA reaction) showing the peaks corresponding to double labeled SP1 with a low intensity after aqueous dilution.

## Slide 2
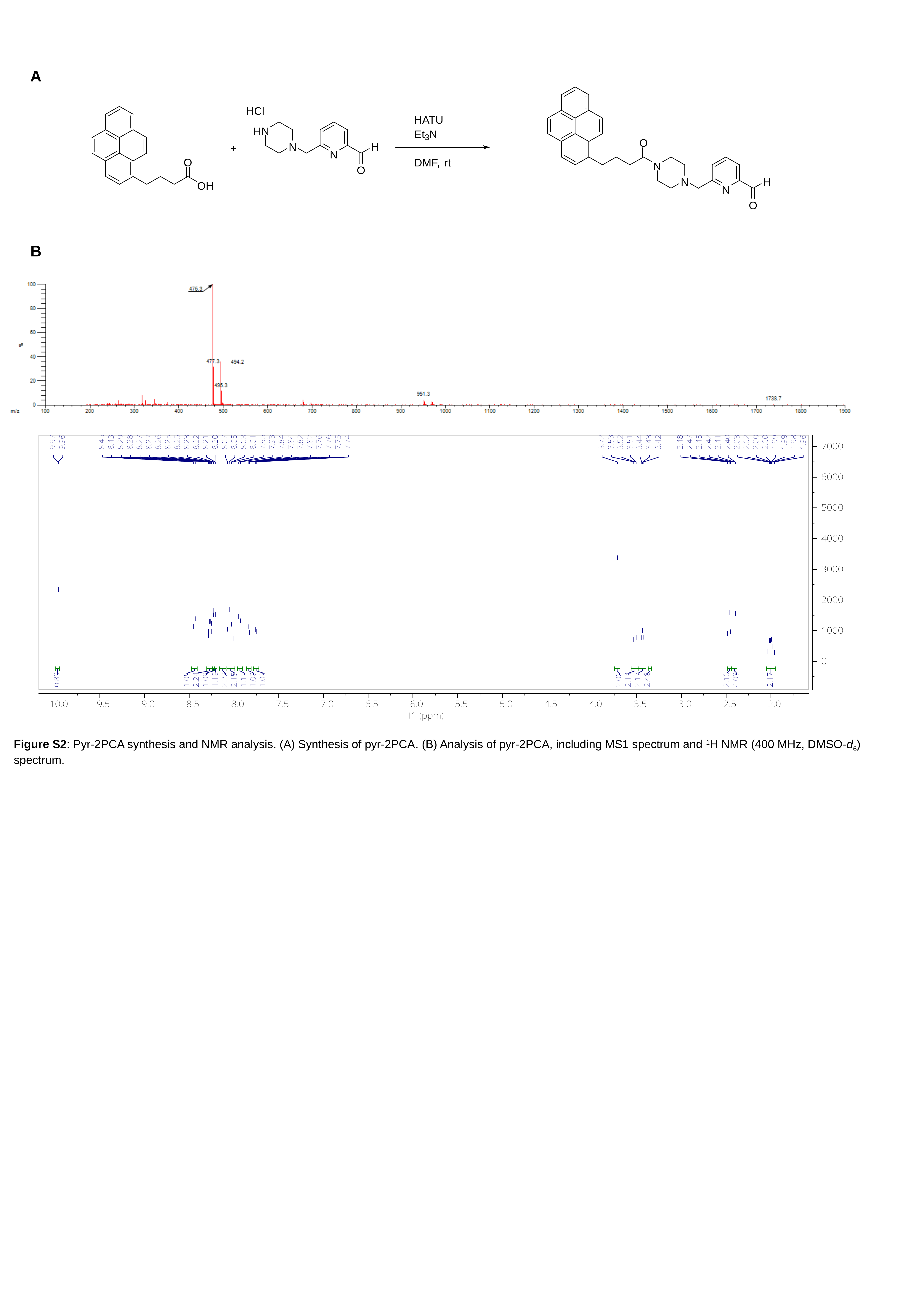

A
B
Figure S2: Pyr-2PCA synthesis and NMR analysis. (A) Synthesis of pyr-2PCA. (B) Analysis of pyr-2PCA, including MS1 spectrum and 1H NMR (400 MHz, DMSO-d6) spectrum.

## Slide 3
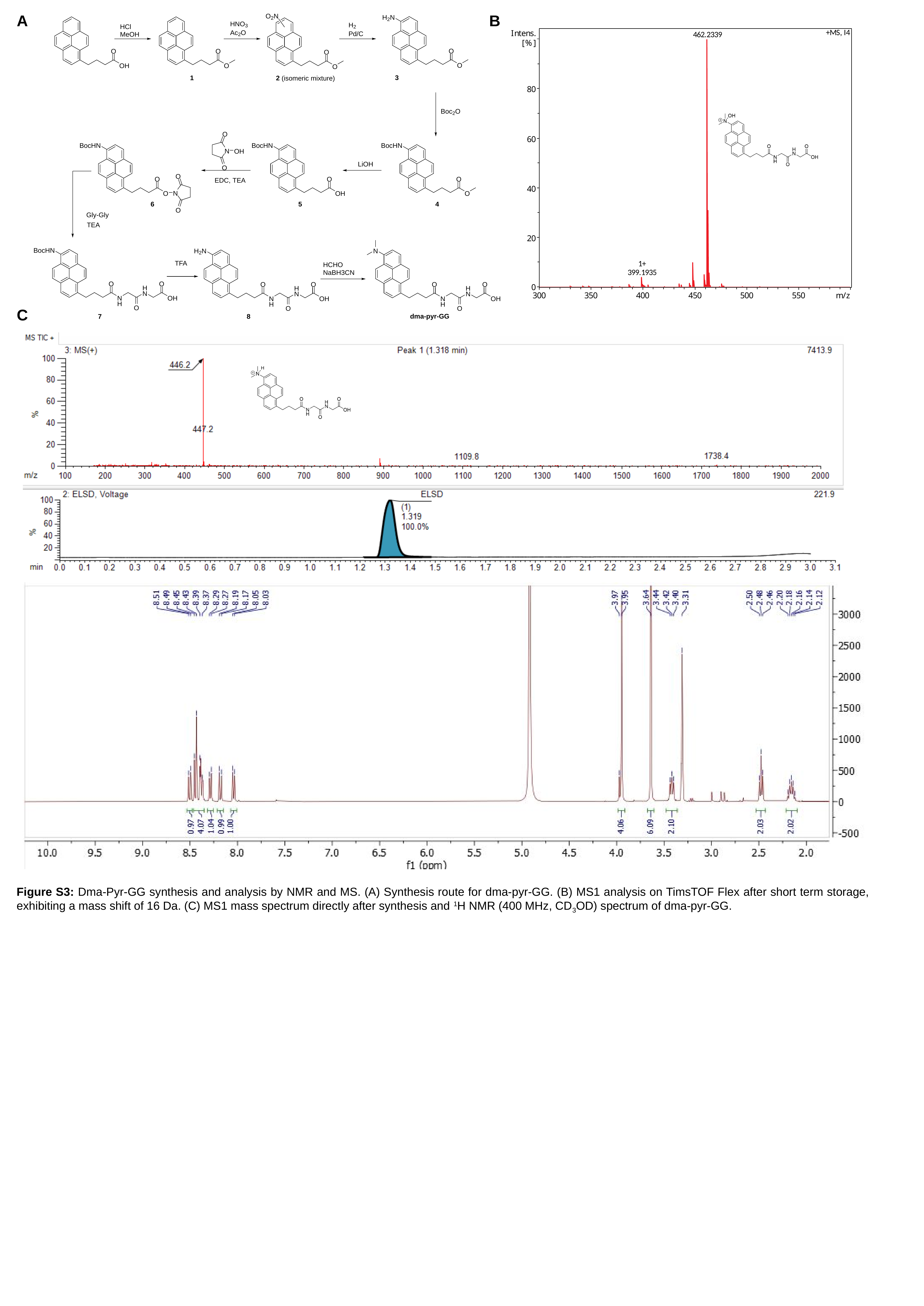

A
B
C
H
Figure S3: Dma-Pyr-GG synthesis and analysis by NMR and MS. (A) Synthesis route for dma-pyr-GG. (B) MS1 analysis on TimsTOF Flex after short term storage, exhibiting a mass shift of 16 Da. (C) MS1 mass spectrum directly after synthesis and 1H NMR (400 MHz, CD3OD) spectrum of dma-pyr-GG.

## Slide 4
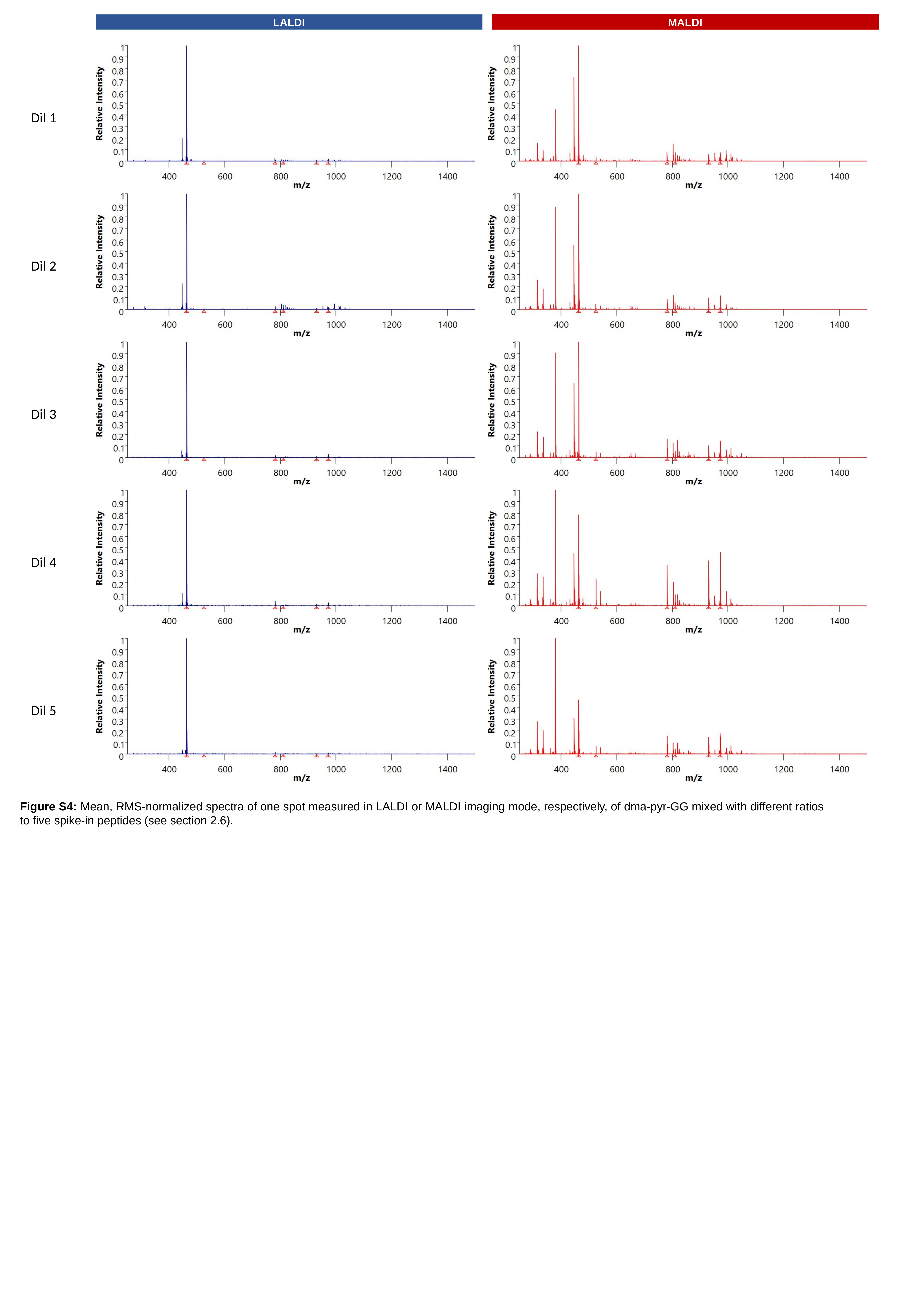

MALDI
LALDI
Dil 1
Dil 2
Dil 3
Dil 4
Dil 5
Figure S4: Mean, RMS-normalized spectra of one spot measured in LALDI or MALDI imaging mode, respectively, of dma-pyr-GG mixed with different ratios to five spike-in peptides (see section 2.6).
